# In situ deep-sea surface-enhanced Raman scattering sensor reveals cyanide as a key factor in cold seep nitrogen cycling

**DOI:** 10.1126/sciadv.aef5836

**Published:** 2026-08-14

**Authors:** Siyu Wang, Tingting Zhao, Wanying He, Fei Li, Zhicheng Wang, Ruhao Pan, Lianfu Li, Shichuan Xi, Zhendong Luan, Zhiyang Zhang, Bowei Li, Lingxin Chen, Xin Zhang

**Affiliations:** ^1^ LaoShan Laboratory, Qingdao 266237, China.; ^2^ Shandong Key Laboratory of Coastal Zone Environmental Processes and Ecological Security, Coastal Zone Ecological Environment Monitoring Technology and Equipment Shandong Engineering Research Center, Yantai Institute of Coastal Zone Research, Chinese Academy of Sciences, Yantai 264003, China.; ^3^ School of Chemistry and Chemical Engineering, Linyi University, Linyi, 276005 Shandong, China.; ^4^ College of Safety and Environmental Engineering, Shandong University of Science and Technology, Qingdao 266590, China.; ^5^ Institute of Physics, Chinese Academy of Sciences, Beijing 100190, China.; ^6^ Key Laboratory of Marine Geology and Environment & Center of Deep Sea Research, Institute of Oceanology, Center for Ocean Mega-Science, Chinese Academy of Sciences, Qingdao 266071, China.

## Abstract

Deep-sea hydrothermal vents and cold seeps, typical ecosystems characterized by abundant carbon but limited nitrogen, support thriving microbial activities. The source of nitrogen has long been a key focus in scientific research. Although cultivation experiments have confirmed the presence of diverse nitrogen-fixing microorganisms, direct in situ evidence has been lacking. Therefore, a deep-sea in situ surface-enhanced Raman scattering sensor has been developed. It can adapt to extreme environment (≥350°C and ≥2000-m depth and LOD < 10^−7^ M) and has excellent deep-sea application potential. Crucially, the sensor achieved the in situ detection of cyanide (-CN) (>5.7 μM) at cold seep, providing direct evidence for an energy-efficient nitrogen fixation pathway in the microbial communities. Concurrently, the gradient detection results indicated that CN was consumed as a microbial “circulating currency.” This finding offers critical in situ evidence for understanding the coupling mechanisms of cold seep carbon, nitrogen, and sulfur cycles, marking a substantial breakthrough in deep-sea sensing technology.

## INTRODUCTION

Hydrothermal vents and cold seeps, known for releasing vast amounts of hydrocarbons such as methane, represent typical environments characterized by “carbon enrichment and nitrogen deprivation.” Conventional views held that this strong nitrogen limitation would constrain microbial activity (*1*–*3*). However, researchers have found a nitrogen-fixing community with rich evolutionary diversity and multiple metabolic capabilities (such as ANME-2 archaea), which effectively alleviated this ecological bottleneck by converting inert nitrogen gas (N_2_) and structurally similar cyanide (-CN) into biologically available nitrogen substances (such as ammonia) through various means (*3*–*5*). Subsequently, Jiang *et al.* (*4*) validated through incubation experiments using ^15^N-labeled tracers that the nitrogen loss flux from surface sediments was estimated at 4.96 to 7.63 Tg N year^-1^, primarily driven by denitrifying microorganisms. He *et al.* (*6*) compared various sampling methods and found that non–in situ sampling (such as Niskin bottles) notably alters the microbial community structure and amplifies genes related to environmental stress, resulting in an underestimation of the true activity of native microorganisms involved in basic elemental cycles such as carbon fixation, ammonia oxidation, and methane oxidation, therefore developing a new generation of sensing technologies capable of withstanding high temperature and pressure while enabling highly sensitive in situ detection, such as deep-sea Raman spectroscopy (*7*–*10*). This is to obtain key in situ evidence for the resistance of deep-sea microorganisms to nitrogen deficiency and to provide technical support for revealing the coupling mechanism of the deep-sea carbon-nitrogen cycle.

Raman spectroscopy, a rapid and nondestructive detection method, can help assess the chemical composition of live microorganisms in a nearly real-time manner and holds great potential for application in microbiology (*11*–*14*). Because of the complexity of the deep-sea environment and the low abundance of extremophiles and their metabolites, conventional Raman spectroscopy is unable to achieve the in situ identification of deep-sea microbes and their metabolic products (*15*). Surface-enhanced Raman scattering (SERS) technology offers notable advantages—including ultrahigh sensitivity, rapid detection, and no-label detection—in the in situ identification of deep-sea microbes metabolites (*16*, *17*). Wang *et al.* (*15*, *18*) designed deep-sea SERS probes achieving a detection limit of 10^−6^ M and capable of withstanding conditions of up to 11 MPa/4°C. However, the extreme environment of the deep sea is characterized by low (≤4°C) or high (≥300°C) temperatures, high pressures, and complex fluid components (*19*), which pose a major challenge to the extreme environmental tolerance and flexibility of traditional SERS substrates (*15*, *18*). Owing to the excellent characteristics—such as light weight, high strength, and high temperature, and corrosion resistance—of carbon fiber cloth (CFC), it could be used as an effective SERS carrier for the in situ detection in deep sea (*20*).

Here, a nondestructive, label-free molecular spectroscopy analysis method was proposed to rapidly obtain the in situ spectral information characteristics of extremophiles and metabolites in deep sea. This method is realized by designing a high temperature–resistant, high pressure–resistant, and flexible deep-sea SERS sensor (D-SERS sensor). The flexible SERS substrate designed in this work constitutes the core of the entire sensor; it can be mounted on an in situ deep-sea Raman probe to enable effective in situ replacement and multipoint detection. This SERS substrate uses the silver (Ag) layer of electron beam deposition (EBD) to provide relatively more stable anchor points for gold (Au) nanoparticles (NPs), demonstrating excellent stability. The CFC has both flexibility and a 3D structure, which endows the D-SERS sensor with the ability to rapidly couple with the deep-sea in situ Raman system. This D-SERS sensor exhibits excellent performance in the detection of various organic molecules and biological metabolites, e.g., nucleotides and others. The high-pressure and high-temperature (HPHT) simulation was performed on the D-SERS sensor to verify its applicability to the extreme conditions of deep-sea hydrothermal fluids. Last, the applicability of this method was validated through on-site and in situ tests in the deep sea and successfully obtaining the Raman spectra of various microorganisms in different regions of the cold seep (symbiotic bacteria, methanogenic archaeon, and others). The D-SERS sensor not only increased the detection limit to <10^−7^ M but also could withstand conditions of ≥350°C/20 MPa and achieved the in situ detection and direct quantification of -CN. Cyanide is not merely a metabolic by-product or environmental toxin as traditionally perceived but is highly likely to play a pivotal role in connecting the carbon, nitrogen, and sulfur cycles, providing insights into the adaptation of deep-sea microorganisms.

It is worth noting that the exploration of the two cold seep sites and the multiple gradient detections were completed in just two diving trips. This was impossible to achieve in previous in situ SERS detection work (due to the difficulty in replacing the SERS substrate and the problem of in situ focusing). This work successfully overcame this challenge and reduced the cost of in situ SERS detection in the deep sea by more than $380,000 [now, each time we carry D-SERS sensors more than 30 pieces. Previously, we could only carry one piece sensor each time. The cost of each remotely operated vehicle (ROV)/ Human-Occupied Vehicle (HOV) dive was at least $20,000].

## RESULTS

### Preparation and characterization of the D-SERS sensor

**Fig. 1.**
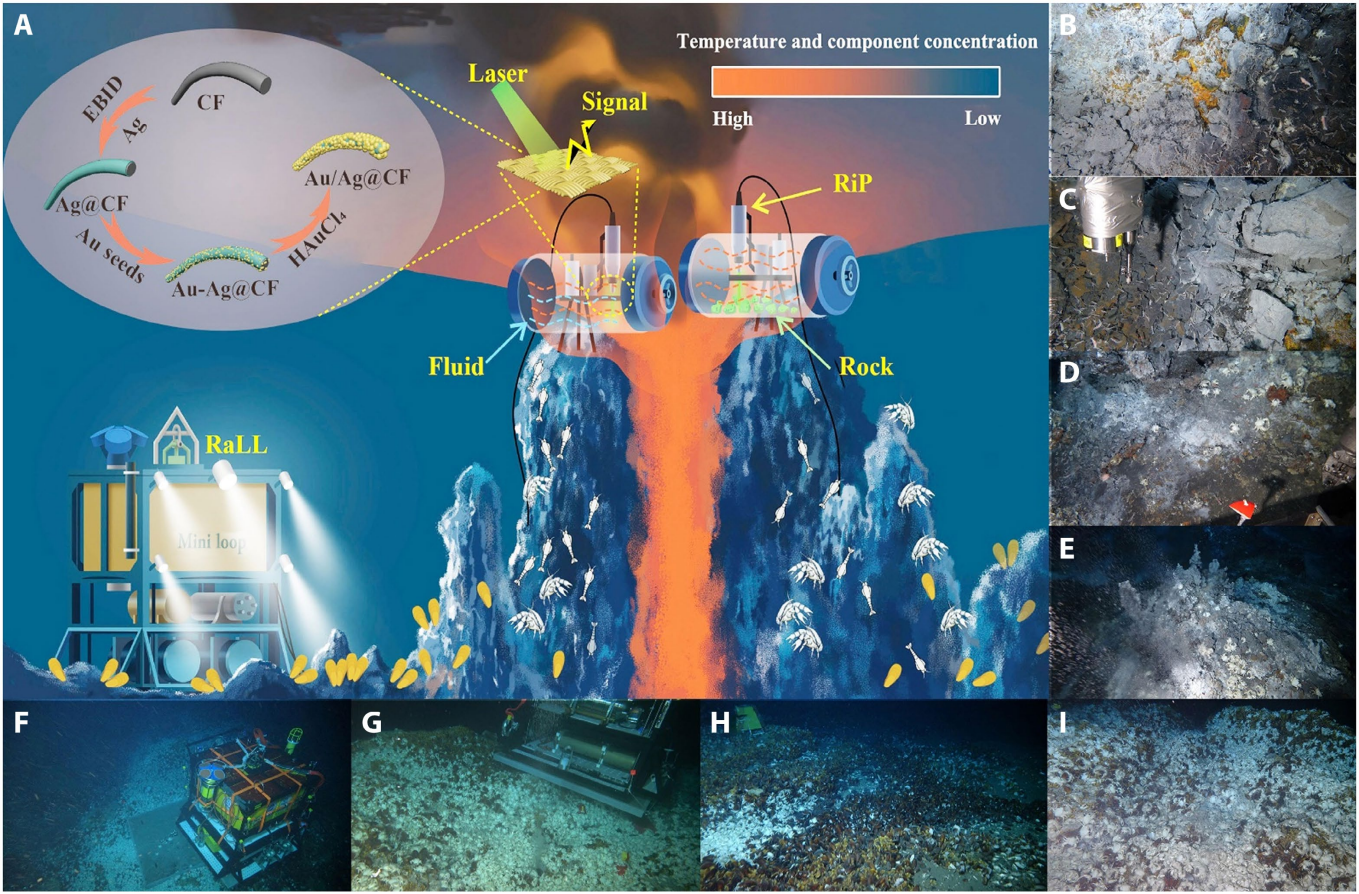
Schematic diagram of the thermal fluid operation using D-SERS probes. (**A**) Schematic diagram depicting the interactions of environmental factors and the distribution of biological community in deep-sea hydrothermal vent area and concept diagram of surface-enhanced Raman scattering (SERS) detection system working mode. Schematic depicting the preparation of the carbon fiber (CF) cloth (CFC)–based SERS substrate loaded with gold (Au) NPs/silver (Ag) layer structure (Au/Ag@CFC) is presented in the top left corner. (**B** to **E**) Organisms in hydrothermal vents. (**F** and **G**) Image of deep-sea lander mini loop. (**H** and **I**) Organisms in cold seep vents.

Figure 1A shows that the environmental factors in the deep-sea hydrothermal zone, with a large amount of inorganic salts, heavy metal ions, and dissolved gases—such as methane, carbon dioxide, and hydrogen—being ejected from the hydrothermal vents interact with the surrounding fluids (*21*, *22*). Moreover, many organisms also live in the hydrothermal zone (Fig. 1, B to E, and figs. S2 and S3) and cold seep (Fig. 1, F to I). The in situ studies of the metabolites of these organisms can provide relatively more accurate evidence of relevant biological interaction factors. Therefore, a D-SERS sensor suitable for high-temperature and high-pressure environments have been designed. The core component of the D-SERS sensor is a high temperature–resistant flexible SERS substrate, and the preparation process is shown in the inset of Fig. 1A (detailed description in the Supplementary Materials). This senser is expected to enable the in situ detection of extremophiles and their metabolites in deep-sea extreme environments.

**Fig. 2.**
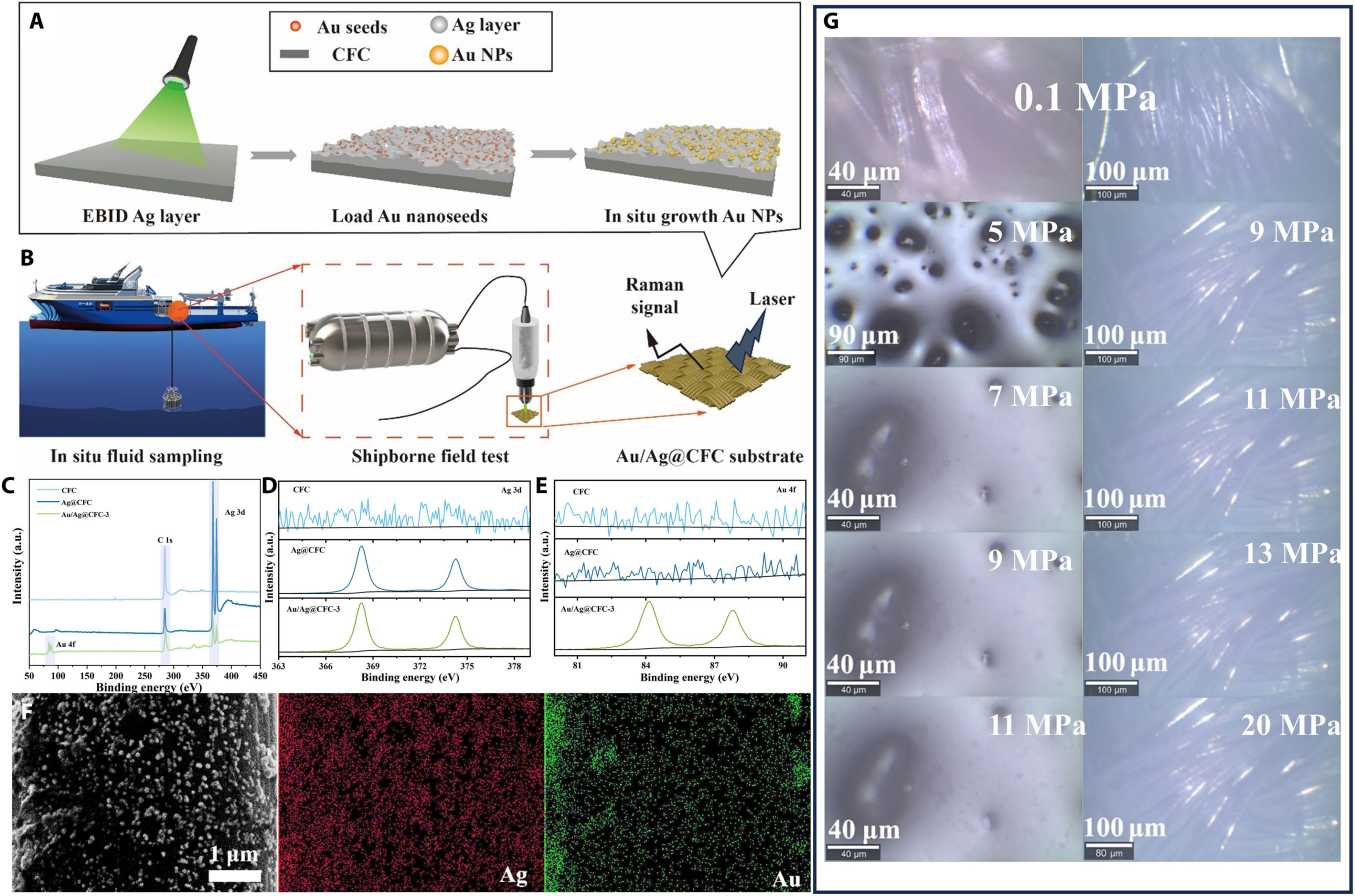
D-SERS probe technology and its advantages. (**A**) Schematic depicting the process used for the preparation of the CFC-based substrate loaded with Au NPs/Ag layer structure (Au/Ag@CFC-3). (**B**) Schematic diagram of the procedure used for the on-site shipborne test of the Au/Ag@CFC substrate. (**C** to **E**) Total (C), Ag 3d (D), and Au 4f (E) XPS spectra of CFC, Ag@CFC, and Au/Ag@CFC-3. (**F**) EDS mapping of Au/Ag@CFC-3 (red dot: Ag; green dot: Au). (**G**) States of cloth (left) and CFC (right) under different pressures (captured using confocal microscopy). a.u., arbitrary unit.

The core of the construction of D-SERS sensor lies in the preparation of high-performance SERS substrates. In this study, first, an Ag layer was deposited on the surface of CFC using EBD technology. The Ag layer can provide binding sites for the loading of Au nanoseeds (*23*). After the adsorption of Au seeds onto the Ag layer surface, the obtained Au seeds/Ag@CFC substrates were immersed in the growth medium to grow Au NPs in situ (Fig. 2A). On the basis of research reports, the plasma performance of Au nanostructures can be enhanced by doping with Ag (*24*, *25*). Therefore, the protrusions on the surface of the Ag layer (shown in Fig. 2F) are expected to further improve the SERS performance of Au NPs. Moreover, owing to the satisfactory three-dimensional structure of CFC itself, the Au/Ag@CFC-3 substrate with 3.0 mM HAuCl_4_ concentration also met the requirements of in situ detection without focusing. Last, it was also verified through on-site tests at sea. The relevant experimental mode is shown in Fig. 2B. The in situ fluids of the cold seeps can be directly detected on-site using the onboard deep-sea sampling equipment or they can be used the D-SERS sensor to the deep sea for in situ real-time detection.

The enhanced Raman effect of D-SERS sensors originates from the noble metal NPs (such as Au NPs) on the substrate surface. Scanning electron microscopy (SEM) can show the loading of Au NPs on the SERS substrate surface during the preparation process. The SEM images revealed that the surface of CFC was relatively smooth (fig. S5A). When an Ag layer was coated, numerous Ag nanoprotrusion structures appeared on the surface (fig. S5B), which could provide suitable sites for the loading of Au seeds. Figure S5F shows large amounts of Au NPs growing on the surface of the Ag@CFC-3 substrate, which indicates the successful synthesis of the SERS substrate. Figure S4 (A to C) presents a low-magnification image, showing the morphology after Ag layer deposition and growth of Au NPs on carbon fibers (images shown in fig. S4, A to C corresponds to those shown in fig. S5, A, B, and F, respectively). X-ray photoelectron spectroscopy (XPS) tests were then performed on the three substrates (Fig. 2, C to E). The presence of Au 4f on the Au/Ag@CFC-3 substrate was observed in the XPS total spectrum. Moreover, the weakening of the peak intensity of Ag 3d may be caused by the masking of the signal by the Au NPs growing on the Ag layer surface. Subsequently, the loading of Au NPs was further verified from the energy dispersive spectroscopy (EDS) mapping images (Fig. 2F). The x-ray diffraction (XRD) analyses were performed further confirmed this result (in the Supplementary Materials).

Notably, CFC-based substrates demonstrated relatively greater stability in the high-pressure environment of water bodies. In our previous study (*21*), the cloth-based SERS substrate underwent notable changes in water under 0.1-, 5-, 7-, and 9-MPa pressure (as shown from top to bottom, respectively, in the left column of Fig. 2G, captured using confocal microscopy). It can be observed that the fabric-based fibers expanded in the water after the pressure increased, which may cause the Au NPs on the fiber surface to fall off or the spacing to increase, thereby affecting the SERS performance. However, the CFC-based SERS substrate maintained a stable state under a pressure of 0.1 to 20 MPa (as shown in the right column of Fig. 2G, captured using confocal microscopy). Furthermore, a comparative analysis of the SEM images revealed that the distribution of Au NPs on the CFC fibers was more uniform than that on the fabric fibers (fig. S6), possibly because the Ag layer uniformly deposited on the surface of the CFC fibers provided more binding sites for the Au NPs. The above facts indicate that the Au/Ag@CFC substrate has greater stability. Therefore, the SERS substrate prepared in this study exhibited superior stability and was found to be relatively more suitable for in situ studies in the extreme environments of the deep sea.

The performance of D-SERS sensors is closely related to the morphology of NPs on the surface of the SERS substrate. Therefore, to obtain the best-performing D-SERS sensors, we optimized and compared the preparation conditions of the SERS substrate. By regulating the concentration of chloroauric acid (HAuCl_4_) in the growth solution, SERS substrates with differing ratios of Au and Ag contents can be obtained, thereby optimizing the performance of SERS substrates. SEM images revealed that with the increase in HAuCl_4_ concentration, the concentration of Au NPs on the fiber surface gradually increased, whereas the exposed Ag layer was gradually masked (fig. S5, C to G). However, the size of Au NPs increased, but their density decreased, with increasing HAuCl_4_ concentrations (reaching 5.0 mM) (fig. S5G), likely because the high concentration of HAuCl_4_ led to the aggregation of Au NPs, thereby affecting the morphological changes. Therefore, it can be inferred from the morphology that when the HAuCl_4_ concentration reached 3.0 mM, the SERS enhancement effect of the Au/Ag@CFC substrate was the most optimal. Although EDS assay results (fig. S5H) revealed that the Au/Ag@CFC-1 substrate exhibited the highest percentage of Au and Ag atoms, this may be because of the large Ag layers exposed at the beginning of Au NP formation, and low Au ion concentration led to the rapid formation of some clusters (square particles). However, when considering the ratio of Au to Ag, the Au/Ag@CFC-3 substrate exhibited optimal performance (fig. S5C). Thus, the optimal density distribution of Au NPs, regular morphology, and narrow gap are all key factors for obtaining a high surface plasmonic resonance effect.

### Performance of the D-SERS sensor

**Fig. 3.**
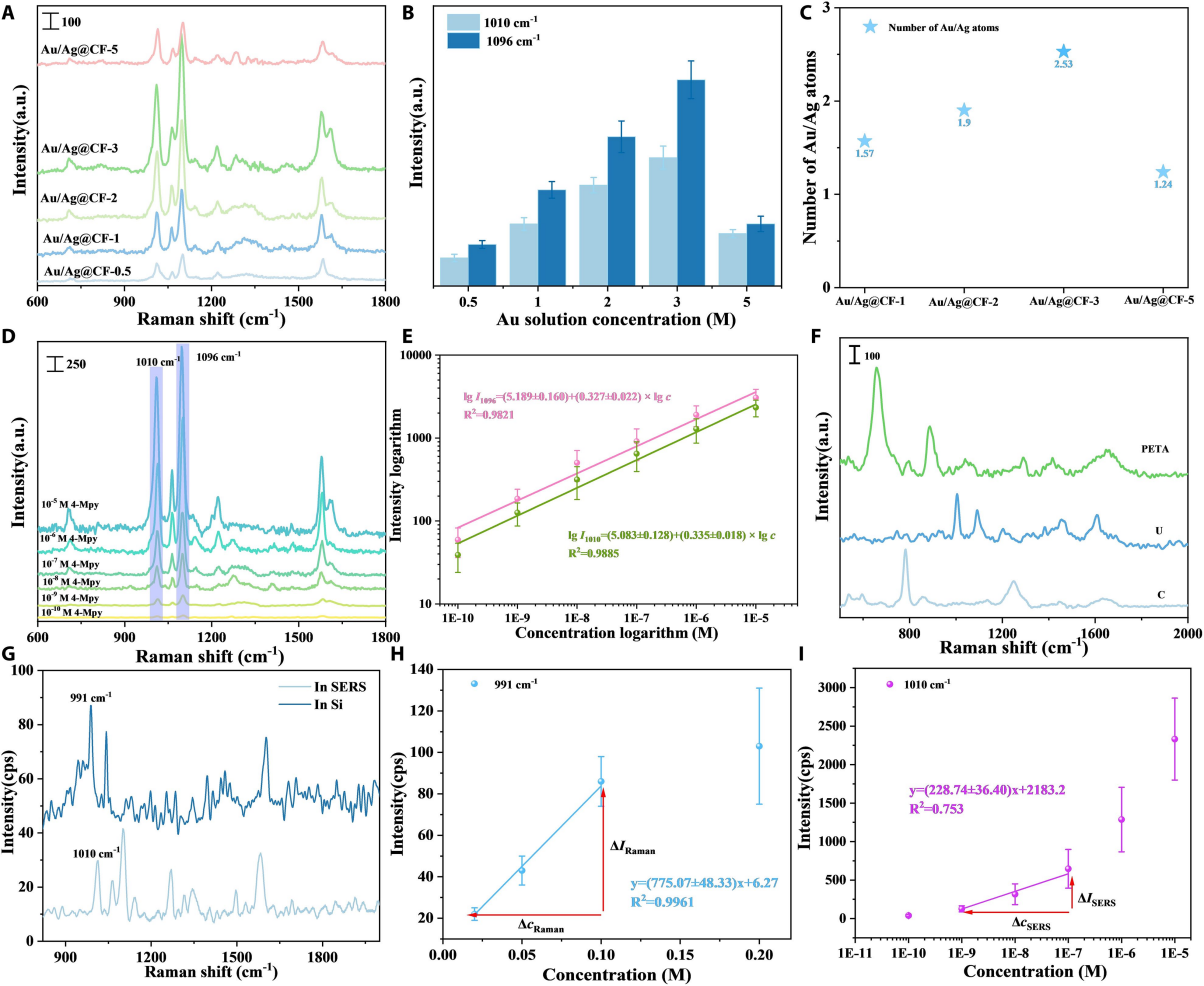
Performance assessment of the surface-enhanced Raman scattering (SERS) substrate. (**A**) SERS signals on substrates loaded with Au NPs/Ag layer structure on CFC, with their chloroauric acid concentrations ranging from 0.5 to 5.0 mM, i.e., on Au/Ag@CFC-0.5 to Au/Ag@CFC-5 substrates, prepared using 4-Mpy (10^−6^ M), with the immersion time being the same for all substrates. (**B**) Au/Ag@CFC-0.5 to Au/Ag@CFC-5 substrate bar chart of Raman peak variation at 1010 and 1096 cm^−1^. (**C**) Ratio of Au atoms to Ag atoms in the Au/Ag@CFC-1 to the Au/Ag@CFC-5 substrate (results correspond to those shown in H). (**D**) SERS spectra of different concentrations of 4-Mpy in the range of 10^−5^ to 10^−10^ M with the same soaking time. (**E**) Intensities of the Raman characteristic peaks of 4-Mpy at 1010 and 1096 cm^−1^ varying from 10^−5^ to 10^−10^ M (logarithmic scale) with varying molecular concentrations (logarithmic scale) corresponding to those shown in (D). (**F**) Diagram depicting the test effect of 10^−6^ M O-phosphorylethanolamine (PETA), uracil (U), and cytosine (C) on the Au/Ag@CFC substrate pair. (**G**) Raman peaks of 0.1 M 4-Mpy on silicon substrates and 10^−10^ M 4-Mpy on Au/Ag@CFC substrates. (**H**) Standard curve between the Raman peak and concentrations of 0.02 to 0.1 M 4-Mpy on the silicon substrates. (**I**) Standard curve between the Raman peak and concentration of 10^−10^ to 10^−8^ M 4-Mpy on the Au/Ag@CFC substrates.

The performance of the D-SERS sensor was initially verified through laboratory tests on the Au/Ag@CFC substrate with respect to the probe molecules (Fig. 3A). The SERS effect was optimal when the HAuCl_4_ concentration was 3.0 mM, i.e., when using the Au/Ag@CFC-3 substrate; then, the Raman intensity decreased notably when the HAuCl_4_ concentration increased to 5.0 mM (Fig. 3B), likely because of the agglomeration of Au NPs at HAuCl_4_ concentrations over 3.0 mM (fig. S5F). The decreased ratio of simultaneously exposed Au/Ag atoms also demonstrated (Fig. 3C and fig. S5G) that relatively more Ag layers were exposed because of the agglomeration of Au NPs, which led to an increase in the distance between NPs and decreased the SERS effect. Therefore, Au/Ag@CFC-3 was selected as the optimal SERS substrate in this study (henceforth referred to as Au/Ag@CFC).

**Fig. 4.**
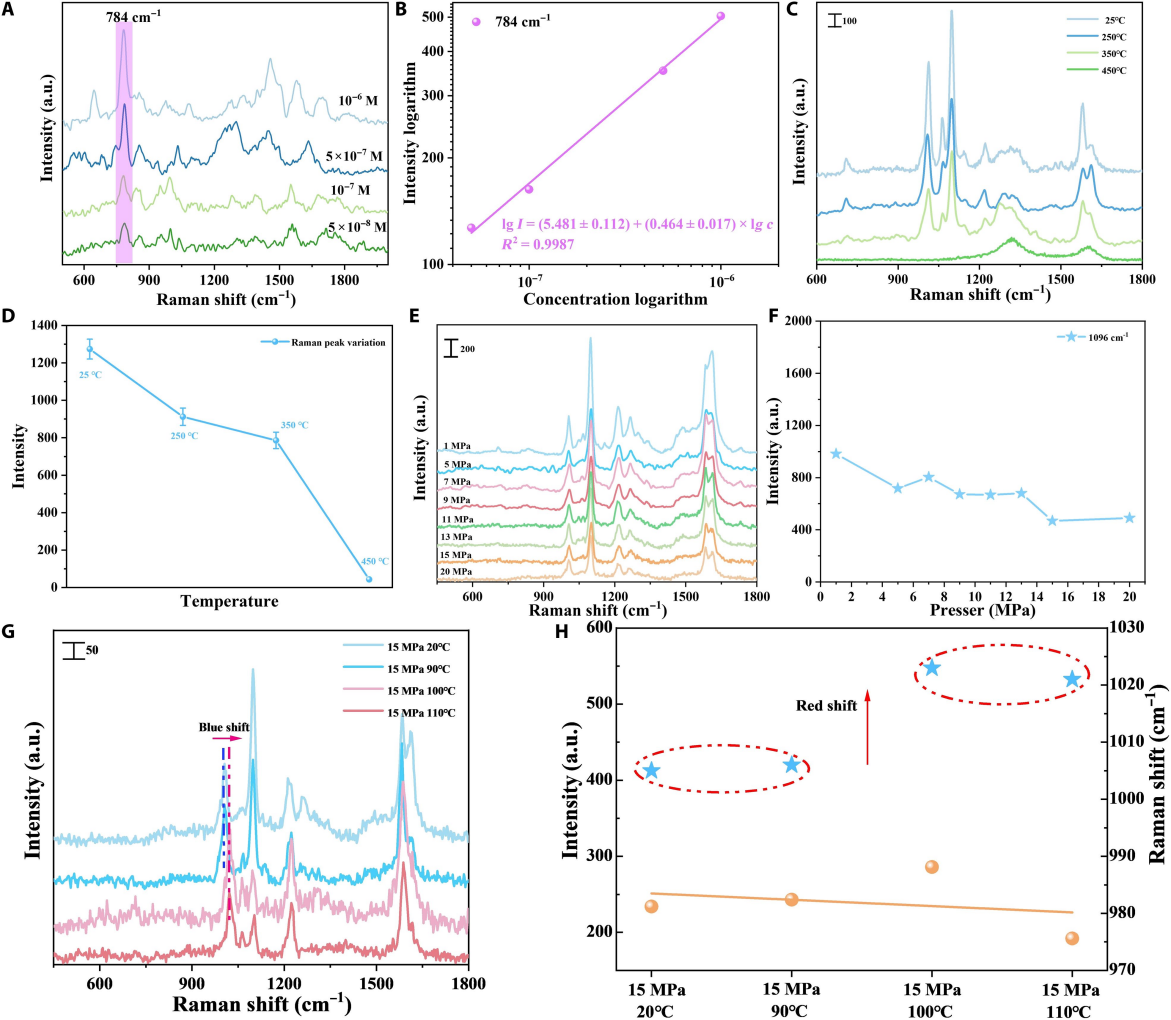
The deep-sea applicability of D-SERS probes. (**A**) SERS spectra of different concentrations of cytosine (C) in the range of 10^−6^ to 5 × 10^−8^ M with the same soaking time. (**B**) Intensities of the Raman characteristic peaks of cytosine at 784 cm^−1^ varying from 10^−6^ to 5 × 10^−8^ M (logarithmic scale) with varying molecular concentrations (logarithmic scale) corresponding to those shown in (A). (**C**) SERS performance tests of the substrate loaded with Au NPs/Ag layer structure on CFC, i.e., the D-SERS sensors, after temperature treatments at room temperature (25°C) and 250°, 350°, and 450°C (with 10^−6^ M 4-Mpy as the signalling molecule); and (**D**) line graph of the characteristic peak changes of the 4-Mpy signal peak at 1010 cm^−1^ on substrates treated at the different temperatures. (**E**) SERS spectra of the D-SERS sensors from under atmospheric pressure to 20 MPa (with 4-Mpy as the probe molecule). (**F**) Line graph of the characteristic peak of 4-Mpy at 1096 cm^−1^ varying with pressure. (**G**) SERS spectra of the D-SERS sensors under simulated temperature and pressure (90° to 110°C, 15 MPa) in deep-sea hydrothermal organisms (with 10^−6^ M 4-Mpy as the probe molecule). (**H**) Peak fitting and Raman shift variation graph of the characteristic peak of 4-Mpy at 1010 cm^−1^ under varying temperatures.

Subsequently, the Au/Ag@CFC-3 substrate served as the SERS substrate, with 4-Mercaptopyridine (4-Mpy) used as the Raman reporter molecule. The Raman spectra of 4-Mpy molecules on the Au/Ag@CFC-3 substrate in the concentration range of 10^−5^ M to 10^−10^ M were obtained to determine the sensitivity of Au/Ag@CFC-3, as shown in Fig. 3D. The results revealed that the 4-Mpy Raman characteristic peaks were present at different positions on the substrate, including the characteristic peaks located at 1010, 1052, 1093, 1208, and 1576 cm^−1^ (*19*). Figure 3E shows the linear relationships between the intensities of the typical 4-Mpy characteristic peaks at 1010 and 1096 cm^−1^ and varying 4-Mpy concentrations. The equation of the red curve (1010 cm^−1^) is lg *I*_1,010_ = (5.189 ± 0.160) + (0.327 ± 0.022) × (lg *c*), and the correlation coefficient is 0.9821. The equation of the green curve (1096 cm^−1^) is lg *I*_1,096_ = (5.083 ± 0.128) + (0.335 ± 0.018) × (lg *c*), and the correlation coefficient is 0.9885. The enhancement factor (EF)_SERS_ of 4-Mpy in the Au/Ag@CFC substrate was estimated to be 5.1 × 10^8^ (Fig. 3G; for detailed EF_SERS_ calculations, see the Supplementary Materials). Furthermore, in this study, we further adopted the method reported by Ren *et al.* (*26*) to calculate the SERS performance factor (SPF). The SPF of the Au/Ag@CFC substrate was calculated as follows: SPF = (Δ*I*_SERS_/Δ*c*_SERS_)/(Δ*I*_Raman_/Δ*c*_Raman_) ≈ Slope_Linear range of SERS_/Slope_Linear range of Raman_ = 3.29 × 10^10^/775.07 = 4.24 × 10^7^ (Fig. 3, H and I). Last, the detection ability of the Au/Ag@CFC substrate for biomolecules present in the extreme deep-sea environments was explored by checking for several organic molecules that may be present in deep-sea cold seep and hydrothermal biota, including the archaeal biomarkers *O*-phosphorylethanolamine (935 cm^−1^) (*27*), U (1000 to 1100 cm^−1^), and C (784 cm^−1^) as examples (Fig. 3F). The results revealed that the Au/Ag@CFC substrate exhibited satisfactory Raman enhancement for these biomolecules. This evidence provides a strong theoretical basis for the application of Au/Ag@CFC substrates in detecting biomolecules in deep-sea cold seeps and hydrothermal fluids. To further verify the ability of Au/Ag@CFC substrates to analyze biomolecules quantitatively, we performed SERS detection using cytosine nucleoside associated with genetic material as the model molecule. The results revealed that the Raman characteristic peak of cytosine nucleoside at 784 cm^−1^ (*28*) stably existed within the range of 5 × 10^−8^ M to 1 × 10^−6^ M (Fig. 4A) and also presented a strong correlation with the change in concentration (Fig. 4B). This result demonstrated that Au/Ag@CFC substrates exhibit excellent quantitative analysis ability for low concentrations of biomolecules, which is expected to enable the in situ quantitative detection of biomolecules in the deep sea.

### Extreme environment simulation test for D-SERS sensors

The fluid temperature of the deep-sea cold seeps is ∼4°C, which causes relatively little interference to the SERS substrate. However, as temperatures at deep-sea hydrothermal vents can reach more than 300°C, although the areas where most organisms live exhibit a much lower temperature (<100°C) (*29*), Raman probes inevitably come into contact with hydrothermal vents during deep-sea operations. Therefore, it is extremely necessary to explore the stability of the developed D-SERS sensors at extremely high temperatures. Thus, the prepared SERS substrate was placed in a muffle furnace and calcined at 250°, 350°, and 450°C for 10 min each (to simulate the temperature when in contact with the hydrothermal vents). To further investigate the stability of these substrates, the holding time was increased to improve the fault tolerance rate. Subsequently, the SERS substrate treated at high temperature was immersed in a 10^−6^ M 4-Mpy solution, adsorbed, and tested, and the corresponding SERS spectra were obtained (Fig. 4C). The results revealed that when the temperature reached 450°C, the Raman signal of only the CFC existed, indicating that both Au and Ag on its surface have been decomposed under high temperatures. However, even after the high-temperature treatment at 350°C, the D-SERS sensors continued to show high SERS performance (Fig. 4D), implying that the D-SERS sensors prepared in this study can withstand the high temperatures of deep-sea hydrothermal environments and is suitable for the in situ detection of microorganisms or their metabolites in deep-sea hydrothermal biological areas and even at the vents.

To further investigate the suitability of the D-SERS sensors in deep-sea hydrothermal environments, HPHT simulation experiments were performed on the D-SERS sensors using a HPHT simulation device (fig. S7) developed by our team. The results revealed that the D-SERS sensors continued to exhibit promising SERS performance even at a high pressure of 20 MPa (∼2000-m depth) (Fig. 4E). It can be seen from Fig. 4F that the signal peak of 4-Mpy has attenuated; however, even at 20 MPa, a relatively strong Raman signal persisted. As mentioned earlier, the temperature in the area where the biological community survives is much lower than that at the nozzle (∼100°C). Therefore, it was necessary to simulate the performance of the developed SERS substrates in the survival environment of the biological community. Here, we simulated the changes in the spectrum of the SERS substrate at 15-MPa pressure (1500-m depth) and 90° to 110°C temperature (with 10^−6^ M 4-Mpy as the reporter molecule) using a HTHP simulation device. It can be observed from Fig. 4G that when the temperature rose to 100°C, the Raman spectrum of 4-Mpy underwent notable changes, and the characteristic peak at 1010 cm^−1^ exhibited a notable blue shift. This result may be attributed to the increase in temperature, which enhances hydrogen bond interactions between water molecules and 4-Mpy, thereby causing the protonation of 4-Mpy molecules, resulting in a blue shift of the Raman peak (pyridine ring respiration vibration) at 1010 cm^−1^ (Fig. 4H, top) (*30*). Although the Raman characteristic peak at 1096 cm^−1^ decreased notably (possibly because of the change in molecular volume) (*31*), the peak value of the characteristic peak at 1010 cm^−1^ did not change notably with the increase in temperature (the peak fitting graph shown in the bottom panel of Fig. 4H), implying that it retains a certain quantitative analysis ability. Furthermore, the long-term stability experiments demonstrated that the D-SERS sensors maintained a notable Raman enhancement effect for over 1 month when stored under sealed conditions (fig. S8). To sum up, the D-SERS sensors designed in this study can be well-applied in deep-sea hydrothermal environments and is expected to enable the in situ detection and quantitative analysis of microorganisms or their metabolites in deep-sea extreme environments.

### Deep-sea application of the D-SERS sensor

**Fig. 5.**
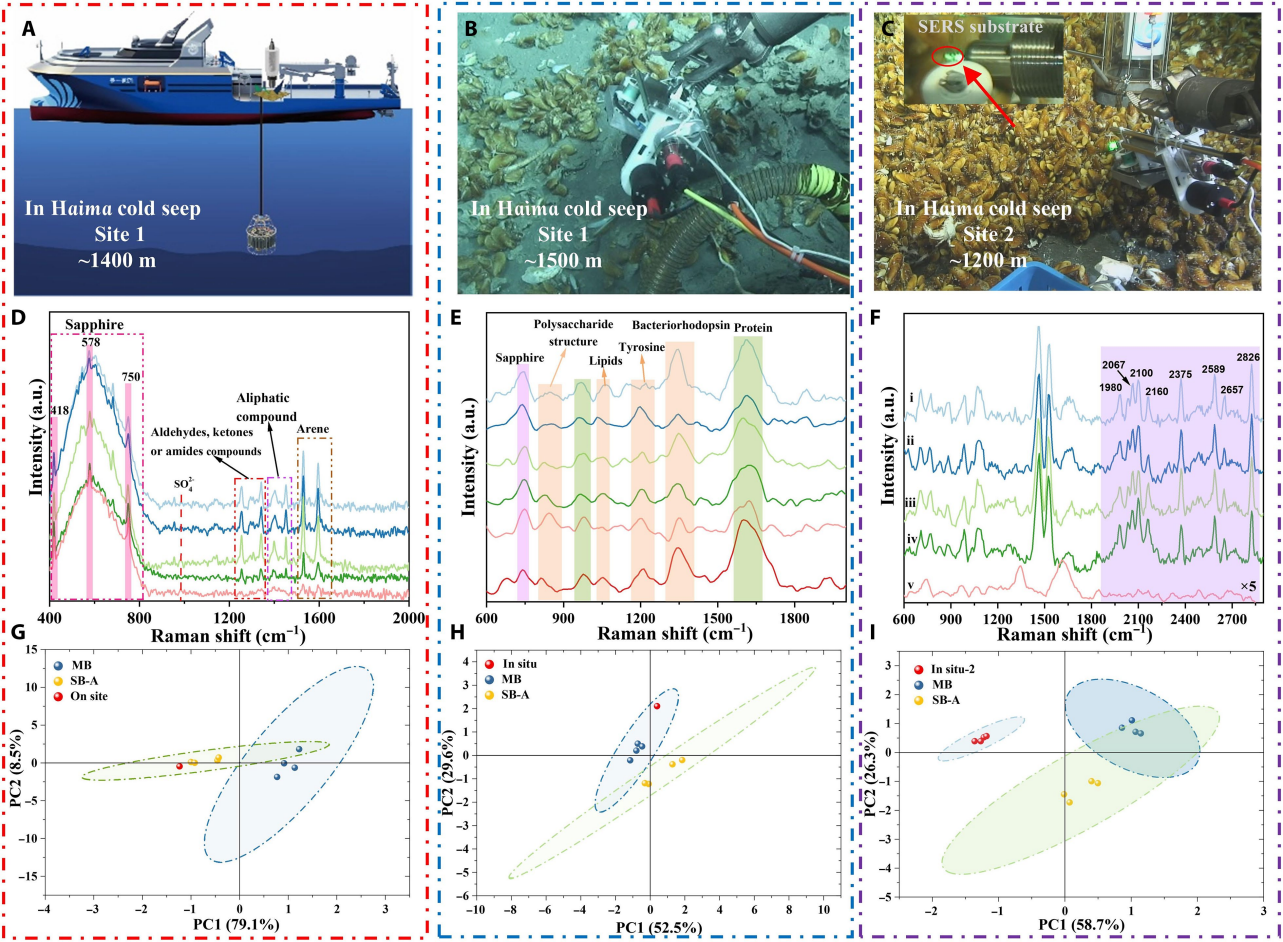
The in situ application of D-SERS probes in the *Haima* cold seep. (**A**) Schematic diagram of the on-site sampling and shipborne testing at the first deep-sea cold seep site 1 (mark as on site). (**B**) Photo of in situ SERS detection at the first deep-sea cold seep site 1 (mark as in situ). (**C**) Photo of in situ SERS detection at the second deep-sea cold seep site 2 (mark as in situ-2). (**D**) SERS spectral data graph obtained from on-site shipborne test. (**E**) SERS spectral data graph obtained from in situ deep-sea test. (**F**) SERS spectral data graph obtained from in situ-2 deep-sea test (i ti iv curve is the SERS spectral of in situ-2 and v curve is the SERS spectral of in situ). (**G** to **I**) Chart showing the principal components (PC) 1 and PC2 scores obtained after performing the PC analysis (PCA) on the Raman spectra and on-site (G), in situ (H), and in situ-2 test (I data of two deep-sea extremophiles, along with the confidence ellipses within the 95% confidence interval. (Note that because of the notable differences in terrain and topography in the deep sea, the depth of cold seeps at different locations can vary greatly).

The actual application at sea can effectively prove the practicability of D-SERS sensor. Three separate sea tests were conducted in the South China Sea (fig. S9): on-site sampling tests at site 1 (the sampling location is ∼100 m above the seafloor, ∼1400-m depth) (ship-based tests), deep-sea in situ tests at site 1 (∼1500-m depth), and deep-sea in situ tests at site 2 (∼1200-m depth) (Fig. 5, A to C). Notably, numerous Raman signal peaks related to biomolecules in cold-seep fluids have been successfully obtained (Fig. 5D). The Raman signals obtained through on-site testing were mainly divided into two regions (Fig. 5D).

First, there were sapphire characteristic peaks within the range of 400 to 800 cm^−1^, mainly derived from the sapphire window of the Raman probe, including the characteristic peaks at 418, 578, and 750 cm^−1^ (*20*). Second, there were biomolecular signal peaks within the range of 800 to 1600 cm^−1^. They can be respectively classified as being located at the characteristic peaks of 1253 cm^−1^ (amide III)^33^ and 1335 cm^−1^ (possibly related to some amino acids) (*32*). The characteristic peaks of 1404 and 1452 cm^−1^ were possibly related to the C─H bending vibration of aliphatic compounds (*33*). Moreover, the characteristic peaks of 1529 and 1597 cm^−1^ were possibly related to the C═C stretching vibration of aromatic hydrocarbons or pigment molecules, such as carotenoids (*34*). Notably, the Raman characteristic peaks of microorganisms have been confirmed to exhibit typical Raman bands at 1235, 1330, 1447, and 1574 cm^−1^ (*35*, *36*). Among them, there are two unique lipid-associated Raman bands containing shear and torsional vibrations of the -CH_2_ and -CH_3_ groups at 1330 and 1447 cm^−1^, which are extremely close to the characteristic peaks at 1335 and 1452 cm^−1^ that we measured (*35*). Moreover, carotenoids have also been confirmed to be present in microorganisms. Therefore, it can be inferred that the collected in situ liquid contains a large number of deep-sea microorganisms. It is worth noting that the sampling depth of the fluid sample was 1400 m, whereas the depth of the cold seep vents was ∼1500 m, and the depth from the bottom was ∼100 m, which indicates that the plumes at the vents carried some microorganisms to areas far from the vents. This observation further suggests that the abundance and spatial distribution of microorganisms must exhibit a strong correlation with the plumes above the vents (*37*, *38*). This further confirms the potential of our designed D-SERS sensor for in situ detection of extreme microorganisms.

Thus, to obtain information regarding the microbes present in the real environment of deep sea. The D-SERS sensor was fixed at the laser focus of the RiP system and carried the Jiaolong manned deep-sea submersible into the Haima cold seep (site 1, ∼1500-m depth) area for in situ detection (the SERS substrate was sealed by two layers of films and opened using mechanical devices after reaching the designated position, and the specific detection method can be seen in Fig. 5B and movie S1). However, the Raman spectra obtained after multiple consecutive spectral acquisitions were different from those obtained in the on-site tests (Fig. 5E). The in situ deep-sea SERS signal peaks were mainly concentrated at 747, 851, 951, 1065, 1199, 1239, and 1609 cm^−1^, which could be attributed to the presence of components such as sapphire, polysaccharide structure, lipid droplets, tyrosine, bacteriorhodopsin, and proteins (table S1) (*39*, *40*). These signal peaks can also be attributed to deep-sea extremophiles or related metabolites. The prominent bacteriorhodopsin-like signals (table S1) are attributed to membrane-associated proteins of chemosynthetic microbes inhabiting the cold seep. We hypothesize that these proteins function as proton pumps driven by chemical energy (e.g., from sulfur or methane oxidation), rather than light, to support microbial survival in the aphotic deep sea. Subsequently, during the second dive (site 2, ∼1200-m depth), we selected an area with abundant organisms (Fig. 5C and movie S2) and conducted in situ SERS detection (denoted as in situ-2). The Raman characteristic peaks obtained here are very sharp, and their intensity is much higher than that of the Raman spectrum obtained at the first position (as shown in Fig. 5F, it is more than five times higher). This indicates that the biological activity here is very active (movie S2 also confirms this observation). The site exhibits substantial methane hydrates and grayish sediments (providing essential substrates for microbial communities such as methanogens and sulfate-reducing bacteria), supporting a notable population of active biological communities, including mussels and their associated symbiotic bacteria. The characteristic peaks within the range of 600 to 1800 cm^−1^ are basically similar to those obtained at the first position (table S2). Ordinary Raman spectra were further collected at this location (fig. S10B). The Raman spectra showed that in addition to the Raman peaks of SO_4_^2−^ and water molecules, there was also a relatively broad fluorescence background peak (speculate that it originates from carotenoids present in microorganisms). This indicates that the signal peaks obtained through the D-SERS sensor are very likely to originate from the deep-sea extremophiles.

Therefore, laboratory-based Raman tests were performed on several deep-sea extremophiles, including symbiotic bacteria (SB-A) from the setae of *Synalpheus crosnieri* and a methanogenic archaeon (MB) from deep-sea cold seep sediments (the strain was derived from the previous work published by our team (*41*)). Figure S10A shows the Raman spectra of the SB-A and the MB and compares them with the on-site, in situ, and in situ-2 deep-sea test results. It was found that the Raman signal peaks detected for the fluid of the cold seep plume were highly similar to those of SB-A, suggesting that SB-A were the dominant species at this location (Fig. 5G). However, the Raman spectra obtained in the in situ deep-sea environment were highly similar to those of the MB, indicating that the MB was the dominant species at this test site (or in the in situ deep-sea environment) (Fig. 5H). The reason for this difference may be that the large organisms, such as amphipods and mussels, inhabiting deep-sea cold seeps mainly inhabit the vent area, with the sediments mostly covered by these large organisms. Therefore, when the cold seep vents erupt, they mainly carry away some of the SB-A and rise with the plume. During in situ deep-sea detection, the biological community is in an apoptotic stage, and the total amount of sediment is much higher than that of the biological population (Fig. 5B and movie S1). Therefore, in the in situ detection results, the main component is the methanogenic archaea present in the sediment. Thus, our work can directly demonstrate that there are notable differences between sampling tests and in situ tests, which further confirm the importance of deep-sea in situ exploration. However, in the second cold spring area, the obtained Raman signals did not match the two deep-sea microorganisms (Fig. 5I). This is because the number of species here is very diverse (Fig. 5C and movie S2), the Raman peak at 2589 cm^−1^ (HS_2_) is relatively high (Fig. 5C), and the Raman peak at 1064 and 1095 cm^−1^ (tetrathionate, in table S1 and fig. S10C) indicates the presence of abundant H_2_S and tetrathionate (*42*). Therefore, it is speculated that sulfur-reducing bacteria constitute the dominant community here (*43*) (the tetrathionate has been confirmed to be an important sulfur source for sulfur-reducing bacteria (*44*)). In conclusion, our proposed method can not only help obtain real in situ information regarding extremophiles inhabiting the deep sea but also provides indications of the metabolic states and biochemical compositions of diverse microbial communities, which has important application value.

### Key evidence of nitrogen fixation behavior of cold seep microorganism

Furthermore, in addition to the typical Raman peaks of biological molecules in the 600- to 1000-cm^−1^ range (possibly originating from deep-sea microorganisms), the in situ-2 spectrogram exhibited many new Raman signal peaks in the 1800- to 2900-cm^−1^ range (Fig. 5F). The signal peaks at 1980, 2067, 2100, and 2160 cm^−1^ was attributed to the stretching vibrations of the C≡N bond in hydrogen cyanide or small-molecule cyano compounds (*45*, *46*). The characteristic peak at 2589 cm^−1^ can be assigned to the S─H bond of proteins (*47*), amino acids, or thiol compounds, while the characteristic peak at 2826 cm^−1^ may originate from the symmetric stretching vibrations of C─H in some lipids or proteins (*48*, *49*). These signals indicate that there is vigorous microbial life activity and complex material and energy exchange here. The discovery of the C≡N signal provides core clues for revealing the deep operational mechanism of this ecosystem.

**Fig. 6.**
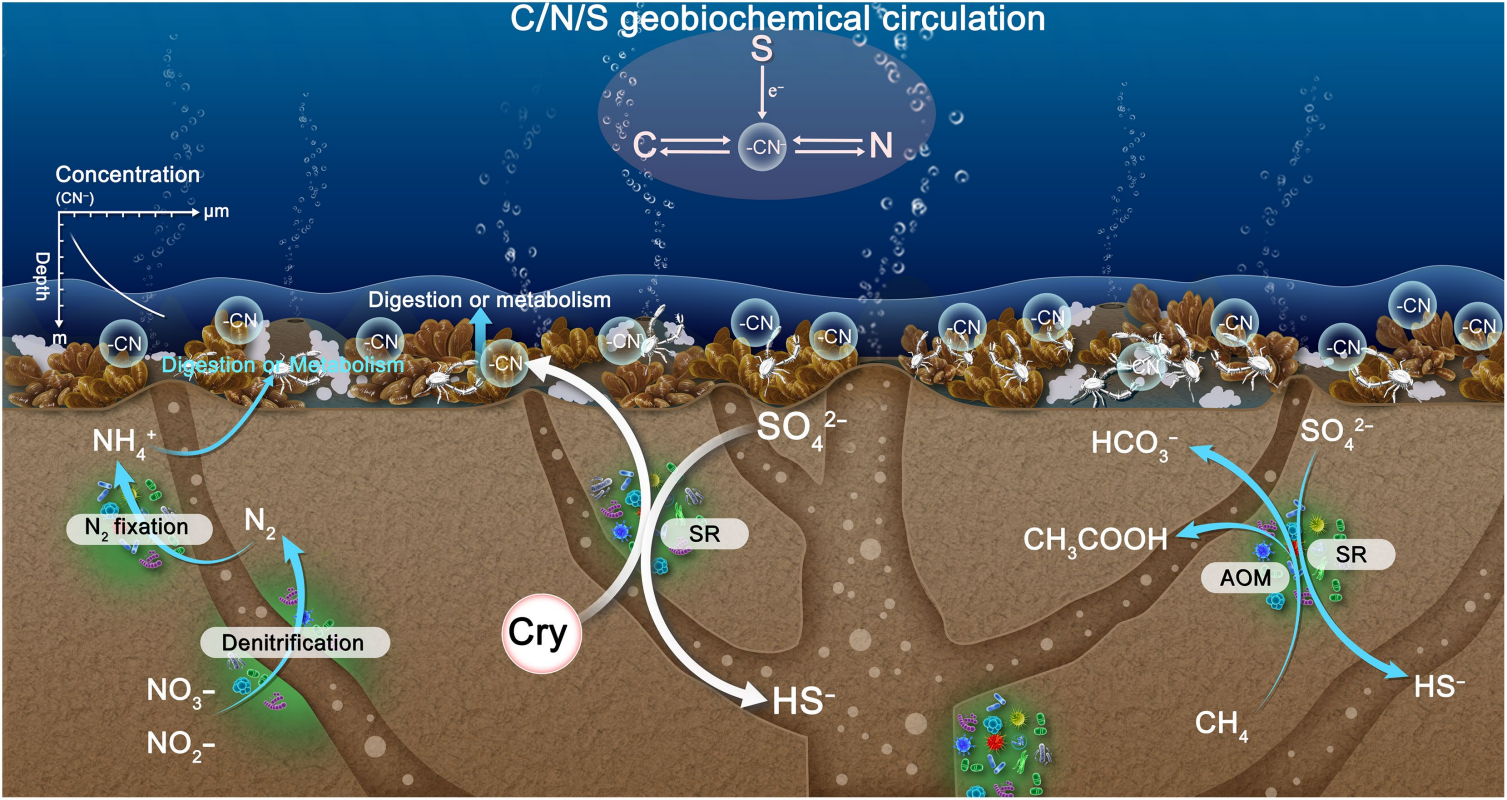
A proposed conceptual model for the carbon, nitrogen, and sulfur cycles in cold seep areas. There are certain microorganisms that can convert glycine to HCN or small molecular cyanide through the cyanoamino acid metabolism (CAM) pathway (from KEGG pathway). Subsequently, -CN (including inorganic cyanides) is metabolized by nitrogen-fixing bacteria (the SR process of sulfur-reducing bacteria can provide energy), which further provides a nitrogen source for the heterotrophic communities in the ecosystem, thereby participating in the elemental cycle of cold seep areas.

This signal may have multiple sources and indicative significance. The work of Dekas *et al.* (*3*) confirmed that nitrogenase, possessed by diazotrophs such as anaerobic methanotrophic archaea (ANME-2), exhibits substrate promiscuity, enabling it to reduce not only N_2_ but also structurally similar cyanide (-CN). Therefore, the presence of the -CN signal serves as direct evidence for in situ nitrogenase activity and further confirms that cyanide can act as an “alternative nitrogen source” for the nitrogen fixation process. Secondly, Kyoto Encyclopedia of Genes and Genomes pathway analysis of metagenomic data revealed that classes such as *Pseudomona*s (common deep-sea bacteria genus) contain complete gene clusters for the glycine metabolic pathway, which is known to produce hydrogen cyanide (HCN) and small molecular cyanides (fig. S11A). This indicates that environmental cyanides are not solely derived from geological sources or as byproducts of fix N_2_; they may also be active synthetic products of microbial metabolism. Last, the co-occurrence of the -CN signal with the S─H (suggesting sulfur metabolism) and C─H (suggesting biosynthesis) signals strongly indicates tight coupling of elemental cycles. Nitrogen fixation requires substantial energy, which likely originates from sulfate reduction (sulfur cycle) coupled with anaerobic oxidation of methane (*5*, *6*). Microorganisms use this energy to fix N_2_ or assimilate cyanide, incorporating the fixed nitrogen into biomass (carbon fixation), thereby achieving deep coupling of the carbon, nitrogen, and sulfur cycles (Fig. 6). Furthermore, the C≡N signal at 2160 cm^−1^ matches the peak height of the signal of the inorganic metal cyanide [Fe(CN)_6_]^4−^. Using the quantitative model established in the laboratory, the concentration of inorganic -CN at this location can be estimated to be ∼5.7 μM (fig. S11, C and D). Therefore, both organic and inorganic sources of -CN exist simultaneously in the cold seep.We also used the actual fluid samples obtained from Haima cold seep as the matrix. We added a standard solution of known concentration of potassium ferrocyanide to this matrix to prepare the spiked sample. Then, we used the D-SERS sensor we developed to detect it and obtained the corresponding Raman spectra (fig. S12). Comparative analysis revealed that, at the same target concentration, the difference in Raman signal intensity measured in the real cold seep fluid matrix compared to that measured in deionized water was less than 10%. This result indicates that the complex matrix components (such as dissolved salts, organic substances, etc.) in the cold seep fluid have limited interference on SERS detection based on the characteristic peaks of C≡N bonds, and the sensor has good stability in its detection of these components.

A long-standing question is whether deep-sea nitrogen fixation solely relies on N_2_ reduction (which is energy-consuming) or uses alternative nitrogen sources. Our in situ data show that there are considerable concentrations of total CN (>5.7 μM) within the cold seep communities, indicating that N_2_ is not the sole substrate for nitrogen fixation. This -CN shortcut may explain why cold seeps can support high microbial biomass even when N_2_ availability is low. Therefore, we propose that cyanide acts as a “circulating currency” in cold seep ecosystems, primarily manifested in its three roles as an efficient energy carrier, a universal nutrient substrate. Also, a key symbiotic mediator, thereby coupling the carbon, nitrogen, and sulfur cycles. First, compared to the highly energy-intensive process of direct microbial nitrogen fixation (reduction of N_2_), assimilating environmental -CN presents a far more economical pathway for nitrogen acquisition, effectively conserving cellular metabolic budget. The -CN constitutes a preformed carbon-nitrogen unit, and its metabolism inherently links the carbon and nitrogen cycles, making it a universal nutrient substrate. Research by Dekas *et al.* (*3*) provides the most direct evidence for cyanide’s “circulating” function, the ANME-2 archaea can not only fix N_2_ but also reduce -CN, sharing the nitrogen-containing products with their symbiotic sulfate-reducing bacteria, thereby acting as a key symbiotic mediator sustaining this cross-domain partnership (fig. S13). Figure S11B shows the almost disappearance of the -CN signal ∼15 cm above the microbial community, implying complete consumption of cyanide within the community without notable diffusion, further corroborating our inferences. Meanwhile, our findings complement those of Jiang *et al.* (*4*), indicating that cold seeps may balance nitrogen loss through gain based on -CN.

### Evidence of the advantages of in situ detection for extremophiles

**Fig. 7.**
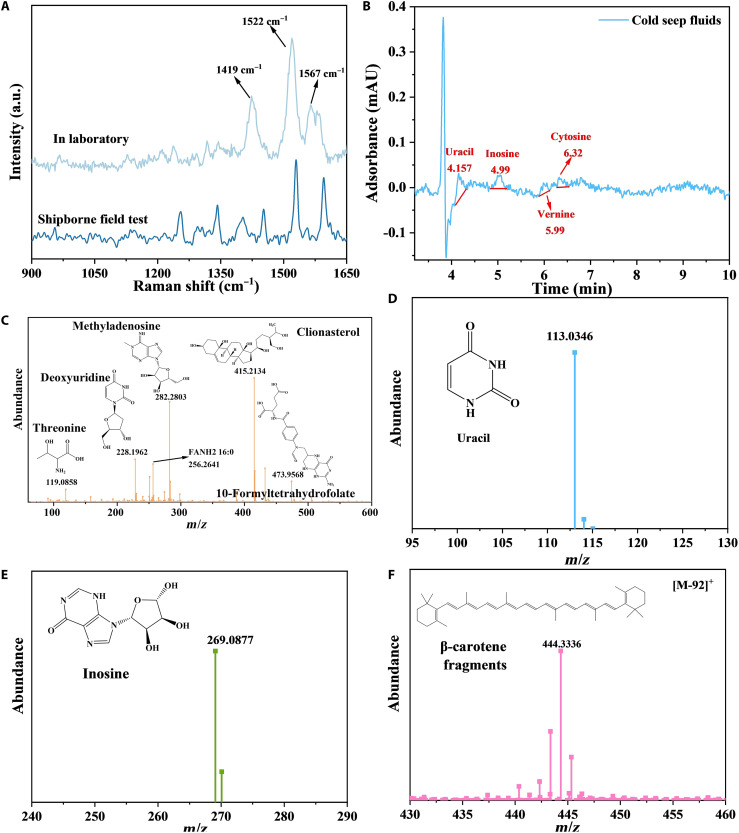
Laboratory analysis of Haima cold seep fluids. (**A**) Raman spectra comparing the SERS test results after transportation back to the laboratory with the on-site test results. (**B**) Liquid chromatogram of the plume fluid from the cold seep vent (filtered only with a 0.22-μm filter membrane). (**C** to **F**) Mass spectrum of the plume fluid collected from the cold seep vent (some mass spectrometry structures were retrieved from the Human Metabolome Database).

After the cold-seep fluid was returned to the laboratory via cold-chain transportation, SERS testing was reperformed on the obtained fluid (Fig. 7A). A comparative analysis of the Raman spectra obtained in the laboratory with those obtained on-site revealed that the fluid composition had changed.

Three relatively notable characteristic peaks appeared in the Raman spectra in the laboratory, located at 1419, 1524, and 1572 cm^−1^. The characteristic peak at 1419 cm^−1^ can be classified as the stretching vibration of the C═C double bond in aliphatic compounds or the ν(C═O) bond in proteins (*50*) (possibly derived from the decomposition of lipids in archaea). The Raman peak at 1524 cm^−1^ measured in the laboratory was abnormally elevated compared with the signal peak of carotenoids (1529 cm^−1^) obtained in situ. This peak may have been produced because of the leakage of carotenoids into the seep fluid after the cell membranes of archaea were broken (or underwent apoptosis). The characteristic peak at 1572 cm^−1^ may have originated from a large amount of DNA-/RNA-based genetic material or nucleotides, such as thymine, uracil, guanine, and adenine (*28*), which may be produced by the decomposition of microorganisms of microorganisms (or archaea). The liquid phase analysis results demonstrated the presence of uracil, adenosine, guanosine, and cytidine in the seep fluid (Fig. 7B and fig. S10D). Furthermore, the mass spectrometry results (Fig. 7, C to F) revealed the existence of biological substances, such as bases, amino acids, carotenoids, various nucleotides, and metabolites (*51*–*53*). The abovementioned facts further indicate that these Raman signal peaks may originate from deep-sea microorganisms. Moreover, the results obtained in this study further validate the significance of in situ testing for deep-sea microbial research and also provide technical and theoretical support for the in situ testing of deep-sea microorganisms in the future.

## DISCUSSION

Biological nitrogen fixation is one of the main sources of available nitrogen for organisms in extreme deep-sea environments, effectively alleviating the nitrogen limitation. This study developed a D-SERS sensor, which can be used for in situ, nondestructive detection of microorganisms and their metabolites in deep-sea extreme environments. Using this sensor, the study demonstrated the presence of a large number of organic compounds in the deep-sea hydrothermal vent microbial community, revealing the rich microbial metabolic activities in this extreme environment. In addition, cyanide compounds were found for the in situ at hydrothermal vents, a breakthrough that provides as perspective for understanding the material cycling of the hydrothermal ecosystem. The cyanide notable enrichment around the microbial community suggests that they may participate in microbial metabolism as intermediate products or alternative nitrogen sources. Particularly noteworthy is the spatial distribution characteristic of cyanide compounds, which shows that they are mainly enriched in the active metabolic areas and sharply decrease 15 cm away from the community, indicating that it may serve as an important “metabolic currency” for transfer between microbial species. This substance transfer mechanism based on cyanide compounds may be a key link in maintaining the carbon-nitrogen-sulfur element coupling cycle of the hydrothermal ecosystem.

The developed D-SERS in situ detection technology in this study provides a powerful tool for the study of microorganisms in deep-sea extreme environments (table S3), while the discovery of cyanide compounds provides a theoretical basis for in-depth understanding of the element cycling mechanism of the chemosynthetic ecosystem in the deep sea. This finding suggests that the cyanide-mediated “energy-saving nitrogen fixation” pathway provides a metabolic advantage in the energy-limited deep-sea environment. Its potential role in driving symbiotic partnerships and niche specialization may have been crucial for the evolution and persistence of complex chemosynthetic ecosystems on Earth and possibly beyond.

However, the D-SERS sensors have two limitations: They are difficult to achieve long-term dynamic collection and unable to distinguish different types of cyanide. The future design will incorporate microfluidic chips to enable long-term in situ observation (supporting deployment for 1 month). And, in the future, through surface modification of specific recognition layers (such as metal complexes targeting free CN^−^ and molecularly imprinted polymers targeting organic cyanide), the sensor will be able to distinguish different forms of cyanide (such as free CN^−^, [M(CN)_6_]^N-^, and organic cyanide compounds), thereby accurately analyzing their sources and transformation pathways in the coupled cycle.

## MATERIALS AND METHODS

### Chemicals and materials

The following materials were purchased from Sinopharm Group (China): HAuCl_4_ 4H_2_O (Au ≥48%), ascorbic acid (AA) (AR, ≥99%), NaOH (≥96%). The following materials were purchased from Aladdin’s reagent platform (China): Polyvinylpyrrolidone (PVP) (molecular weight of 10,000), phosphoethanolamine (≥98%), uracil nucleoside (U) (≥99%), cytosine nucleoside (C) (≥99%), l-phenylalanine (Phe) (≥98.5%), and glutamate (Glu) (≥99%). The following materials were purchased from Macklin (China): 4-mercaptopyridine (4-Mpy) (≥96%), glutathione (reductive, ≥99%), and carbon fiber cloth (CFC) to buy Jingdong shopping platform.

### EBD preparation Ag@CFC

CFC was ultrasonic cleaned in ethanol and aqueous solution for 5 min and vacuum dried at 60°C. A clean CFC substrate is loaded into the chamber of an EBD system (EBD FU-12PEB), and a silver film is then deposited on the substrate at a rate of 2 A/s. The thickness of the film was measured by a thickness monitor, and the silver film with a thickness of 100 nm was obtained by EBD process.

### Synthetic Au/Ag@CFC SERS substrate

The synthesis of gold seeds is adapted from a method reported in the literature. One milliliter of PVP (1 weight %), 100 μl of AA (0.1 M), and 100 μl of NaOH (0.2 M) were mixed, and a tablet with a side length of about 0.5-cm Ag@CFC was added, shook, and adsorbed for 15 min. Then, 100 μl of chloroauric acid (2 mM) was added and shook in the dark for 3 hours. The Au seeds/Ag@CFC was removed and dried. The Au seeds/Ag@CFC was placed in clean petri dishes, dripped with 200 μl of chlorauric acid to react with Au seeds/Ag@CFC for 15 min, remove and dried, soaked in 0.1 M AA solution for 15 min for reduction and removed and dried. The resulting final products were marked Au/Ag@CFC-0.5, Au/Ag@CFC-1, Au/Ag@CFC-2, Au/Ag@CFC-3, and Au/Ag@CFC-5 by immersion of 0.5, 1, 2, 3, and 5 mM chlorauric acid, respectively. After exposure to air for 24 hours, the samples were sealed stored for future use.

### System integration of D-SERS sensors

The D-SERS sensor core component is the Au/Ag@CFC substrate that we fabricated. In addition, the sensor comprises a SERS-substrate transfer tape, a reference platform, a translation stage, and a fixation stage. Unlike our previous report, we have eliminated the detection chamber and adopted an in situ detection approach in which the device is directly immersed in the water column. The D-SERS sensor can be mounted onto the probe of a deep-sea in situ Raman system; the distance between the SERS substrate and the laser focal point can be adjusted to achieve optimal SERS performance. The D-SERS sensor is sealed and stored in a “sandwich” structure between two layers of adhesive tape before installation in the in situ Raman system to prevent seawater contamination and oxidative deactivation in air. Before detection, a rotating mechanism releases the D-SERS sensing unit to be tested and positions it near the focal point for measurement (movie S3).

### Performance of the D-SERS sensor test

The fabricated Au/Ag@CFC substrate served as the SERS platform, with 4-Mpy acting as the Raman reporter to evaluate its enhancement capability. Each substrate was immersed in a 10^−6^ M 4-Mpy solution for 2 hours, followed by air-drying. Raman mapping (Fig. 4A) was then performed to identify the optimal substrate exhibiting maximal sensitivity.

### Instruments

SERS measurements were performed using a DXR Raman microscope (Thermo Fisher Scientific, USA) equipped with a 785-nm laser, which was focused onto the sample via a 50× objective at 25 mW power with a 1-s integration time. Complementary high-pressure analog tests were conducted on a WITec Alpha300 R confocal system. Morphological characterization was carried out using a Hitachi S-4800 field-emission SEM. A 532-nm laser was focused on the sample solution using a 20× microscope. The laser power was 5 mW, and the acquisition time of one spectrum was 1 s. The deep-sea high temperature and high-pressure simulation device was independently developed by the team. The HPLC test uses the U3000 liquid chromatograph from Thermo Fisher Scientific. The liquid chromatography–mass spectrometry test uses liquid chromatography Agilent 1290 UPLC and mass spectrometry Agilent QTOF 6550. The corresponding test conditions are provided in the Supplementary Materials.

Deep-sea Raman insertion Probe (RiP) system: The RiP system comprises four key modules: a primary titanium pressure housing, a probe (integrated with a D-SERS sensor), a hydraulic filtration unit, and oil-filled optical fibers. A complete Raman spectrometer is sealed inside the Ti pressure housing, rated for 4500-m water depth. The oil-filled fibers deliver the excitation light and return the Raman signal. Instrumentation includes a custom NRXNE-532-RA-SP spectrometer and a frequency-doubled Nd:YAG laser (532 nm, 150 mW), both supplied by Kaiser Optical Systems Inc. (USA). An ADU-440A-BV-136 back-illuminated charge-coupled device (CCD) camera (Andor Technology, UK) with 2048 by 512 pixels (27.6 mm–by–6.9 mm active area) is integrated in the housing. The spectral window (100 to 4325 cm^−1^) is mapped onto two CCD segments (100 to 2100 cm^−1^ and 2100 to 4325 cm^−1^), yielding an effective spectral resolution of ∼1 cm^−1^. Before each deployment, wavelength and intensity calibrations are performed with Ne and halogen lamps.

### Application method of D-SERS sensors

First, we conducted a shipborne field on site test on the D-SERS sensor to evaluate its potential for in situ applications in the deep sea. During the May 2025 voyage, the in situ deep-sea RiP developed by the team was loaded onto the ROV. As shown in the red box shown in fig. S14, the front side is the probe and optical cable part of the RiP, and the tail side is the main cabin of the RiP system. The shell is made of titanium alloy and can withstand the water pressure at 4500 m (fig. S14) (the equipment and markings of other units have been blurred). Figure S15 shows an enlarged image of the RiP, with the modified RiP-SERS probe at the top. Compared with the previously designed RiP-SERS probe, the filter head at the front end was removed in the modified probe. Owing to force majeure, the ROV was unable to submerge in the target area again. Therefore, after the in situ fluid above the Haima cold seep area (∼1400-m depth) was obtained by a conductivity-temperature-depth system (SeaBird-SBE911), it was immediately injected into the modified RiP-SERS probe cavity, and on-site tests were conducted on the ship (Fig. 5A). After adsorption for 4 min, real-time spectroscopy was performed using the in situ deep-sea Raman spectroscopy system (the time interval between fluid collection and spectral collection is ∼40 to 60 min). The test interval was ∼1 min, the integration time was 3 s, the number of integrations was three times, and the laser intensity was 87 to 89 mW. Figure S16 shows the photos of some of the biomes in the cold seep vent area.

Deep-sea in situ application: The in situ deep-sea RiP system was installed at the front tray of the “Jiaolong” manned deep-sea submersible (fig. S17). The SERS substrate was fixed at the focal point of the laser window (for details, see the D-SERS sensor design schematic diagram, fig. S1) (sealed to avoid contamination during the descent stage). The test began upon reaching the designated location of the cold seep. The test interval was ∼1 min, the integration time was 1 s, the number of integrations was three times, and the laser intensity was 87 to 89 mW.

### Identification of *hcnABC* genes in MAGs from global cold seeps

Species-level nonredundant representative metagenome-assembled genomes (MAGs) were obtained from a comprehensive genomic catalog of global cold seeps reported by Han *et al.* (*54*). Protein-coding genes were predicted from MAG contigs using Prokka (*55*), and their functions were annotated using the Pfam database (*56*). Putative *hcnA*, *hcnB*, and *hcnC* genes were identified based on functional annotations, and the copy numbers of each gene were summarized separately for each MAG. A phylogenomic tree of MAGs carrying putative hcn genes was constructed using a concatenated alignment of 120 bacterial marker proteins generated with the identify and align modules of GTDB-Tk under default settings (*57*). Phylogenetic inference was performed using FastTree 2 (v2.1.11) (*58*), and the resulting tree was visualized on the iTOL web server (https://itol.embl.de/). To examine the genomic organization of *hcnABC* loci, contigs carrying putative *hcn* genes were extracted and inspected. Genomic neighborhoods surrounding representative *hcnABC* loci were visualized using gene coordinates and strand information derived from Prokka annotations.

## Supplementary Materials

### The PDF file includes:

Supplementary Text Figs. S1 to S17 Tables S1 to S3 Legends for movies S1 to S3 References
