# Supplementary material for: In situ deep-sea surface-enhanced Raman scattering sensor reveals cyanide as a key factor in cold seep nitrogen cycling
Source: Sci Adv. 2026 Aug 14;12(33):eaef5836. doi: 10.1126/sciadv.aef5836 (PMC13475640; doi:10.1126/sciadv.aef5836)
Supplement: Supplementary file 1 [file sciadv.aef5836_sm.pdf]

Science Advances
AAAS

Supplementary Materials for

In situ deep-sea surface-enhanced Raman scattering sensor reveals cyanide as a key factor in cold seep nitrogen cycling

Siyu Wang *et al.*

Corresponding author: Bowei Li, bwli@yic.ac.cn; Lingxin Chen, lxchen@yic.ac.cn; Xin Zhang, xzhang@qdio.ac.cn

Sci. Adv. **12**, eaef5836 (2026)
DOI: 10.1126/sciadv.aef5836

The PDF file includes:

Supplementary Text
Figs. S1 to S17
Tables S1 to S3
Legends for movies S1 to S3
References

Other Supplementary Material for this manuscript includes the following:

Movies S1 to S3

Supplementary Text

The laboratory analysis of the cold seep fluids in this study employed HPLC tests and LC-MS tests. The specific operation process is as follows:

HPLC test:

1. The sample information

    Sample status: colorless solution

    Sample quality: bottled

    Test content: composition analysis

2. Instruments

    U3000 liquid chromatograph, Thermo Fisher Scientific, LTD

3. Experiments and Methods

3.1. Sample processing

    Direct injection testing

3.2 Instrument Conditions

Chromatographic column: XDB-C18 (250*4.6 mm,5 μm);

moving phase:

0 min Water:Acetonitrile = 98 : 2;
3 min Water:Acetonitrile = 98 : 2;
3.1 min Water:Acetonitrile = 97 : 3;
6 min Water:Acetonitrile = 97 : 3;
6.1 min Water:Acetonitrile = 96 : 4;
9 min Water:Acetonitrile = 96 : 4;
9.1 min Water:Acetonitrile = 93 : 7;
17 min Water:Acetonitrile = 93 : 7;
17.1 min Water:Acetonitrile = 98 : 2;
20 min Water:Acetonitrile = 98 : 2;

Flow rate: 1 mL/min;

Column temperature: 30 ℃;

Test wavelength: 260 nm;

Sample size: 10 uL.

LC-MS test: (The solution was subjected to desalination treatment)

Instruments

Liquid chromatography: Agilent 1290uplc

Mass spectra: Agilent qtof6550

Mobile Phase A: 0.1% formic acid aqueous solution; Mobile Phase B: Acetonitrile solution;

0 min Mobile Phase A:Mobile Phase B = 98 : 2;
3 min Mobile Phase A:Mobile Phase B = 98 : 2;
4 min Mobile Phase A:Mobile Phase B = 8 : 92;
5 min Mobile Phase A:Mobile Phase B = 8 : 92;
5.5 min Mobile Phase A:Mobile Phase B = 98 : 2;
7 min Mobile Phase A:Mobile Phase B = 98 : 2;

Flow rate: 0.3 ml/min

The injection volume is 5 μL

Chromatographic column: waters BEH C18 2.1*100 mm 1.7 μm

Mass spectrometry scanning range: 50-600 m/z for the first stage

Sheath gas temp 350 ℃

sheath gas flow 12 L/min
ESI+ mode: Voltage 4000 V

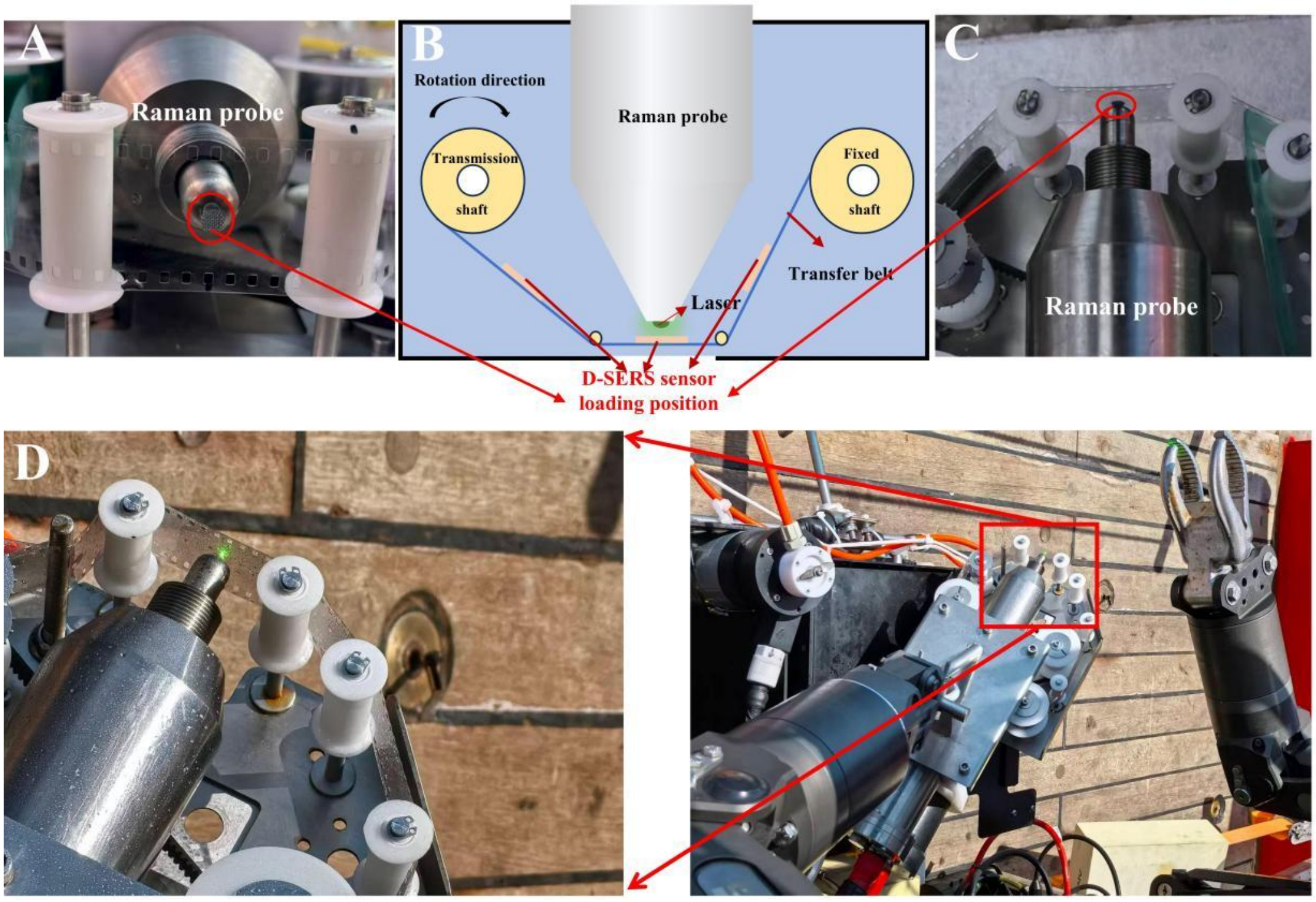

Figure S1 D-SERS Sensors. (A) Side view of the integrated diagram of the D-SERS sensor in the deep-sea Raman detection system. (B) Schematic diagram of the integration for D-SERS sensors in the deep-sea Raman probe system (consisting of components such as the SERS substrate, SERS substrate transfer belt, reference platform, transmission shaft and fixed shaft, etc.). (C) The top view of the integrated diagram of the D-SERS sensor in the deep-sea Raman detection system. (The red circle indicates the actual installation position of the D-SERS sensor). (D): The D-SERS sensor is mounted on the ROV's mechanical arm.

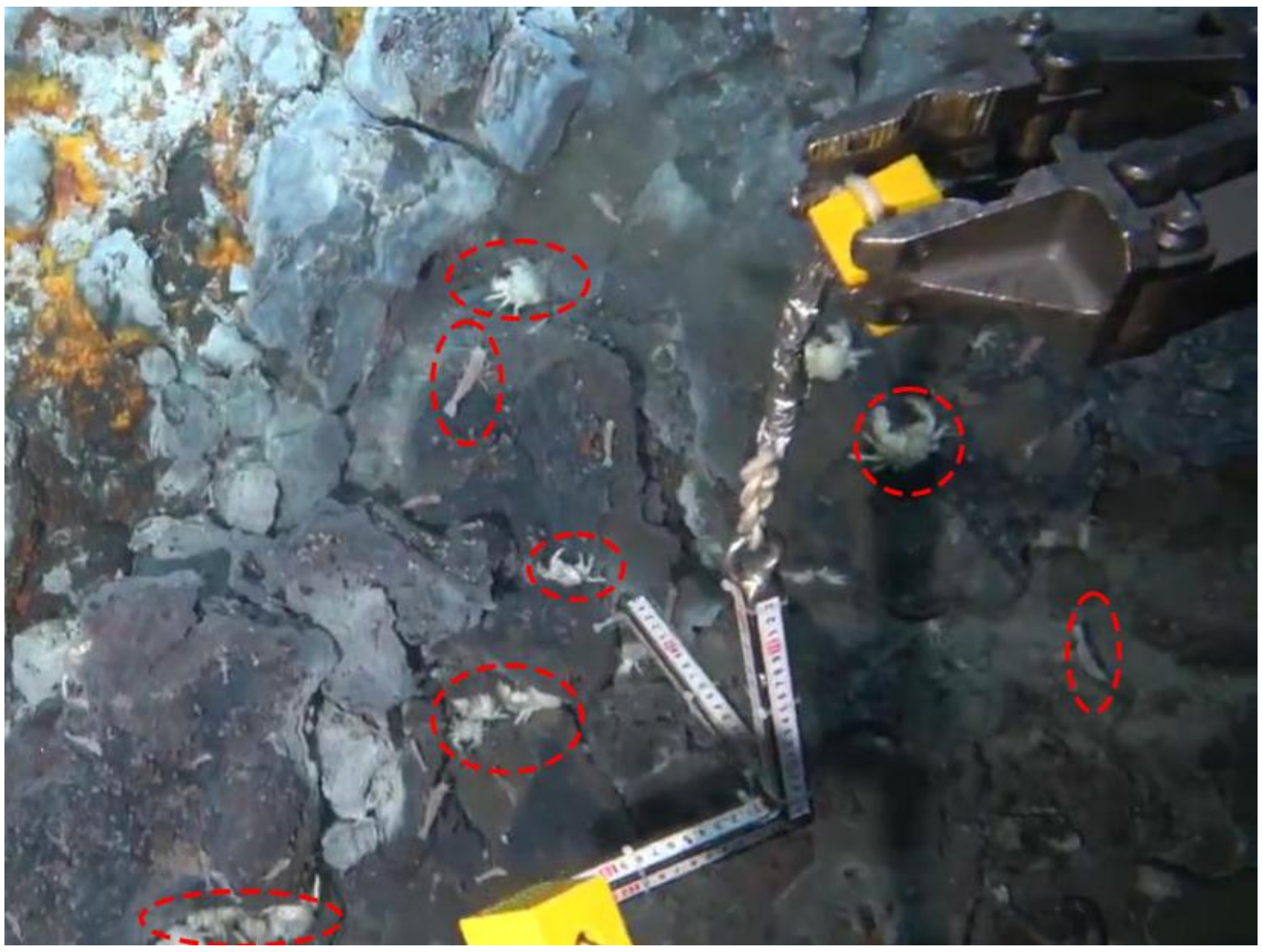

Figure S2 Hydrothermal organisms 1. The photo was taken by the "*Faxian*" ROV in June 2022, in the hydrothermal area of Okinawa (~2000 m depth). A few *Shinkaia crosnieri* (the red circled part) are distributed in the picture.

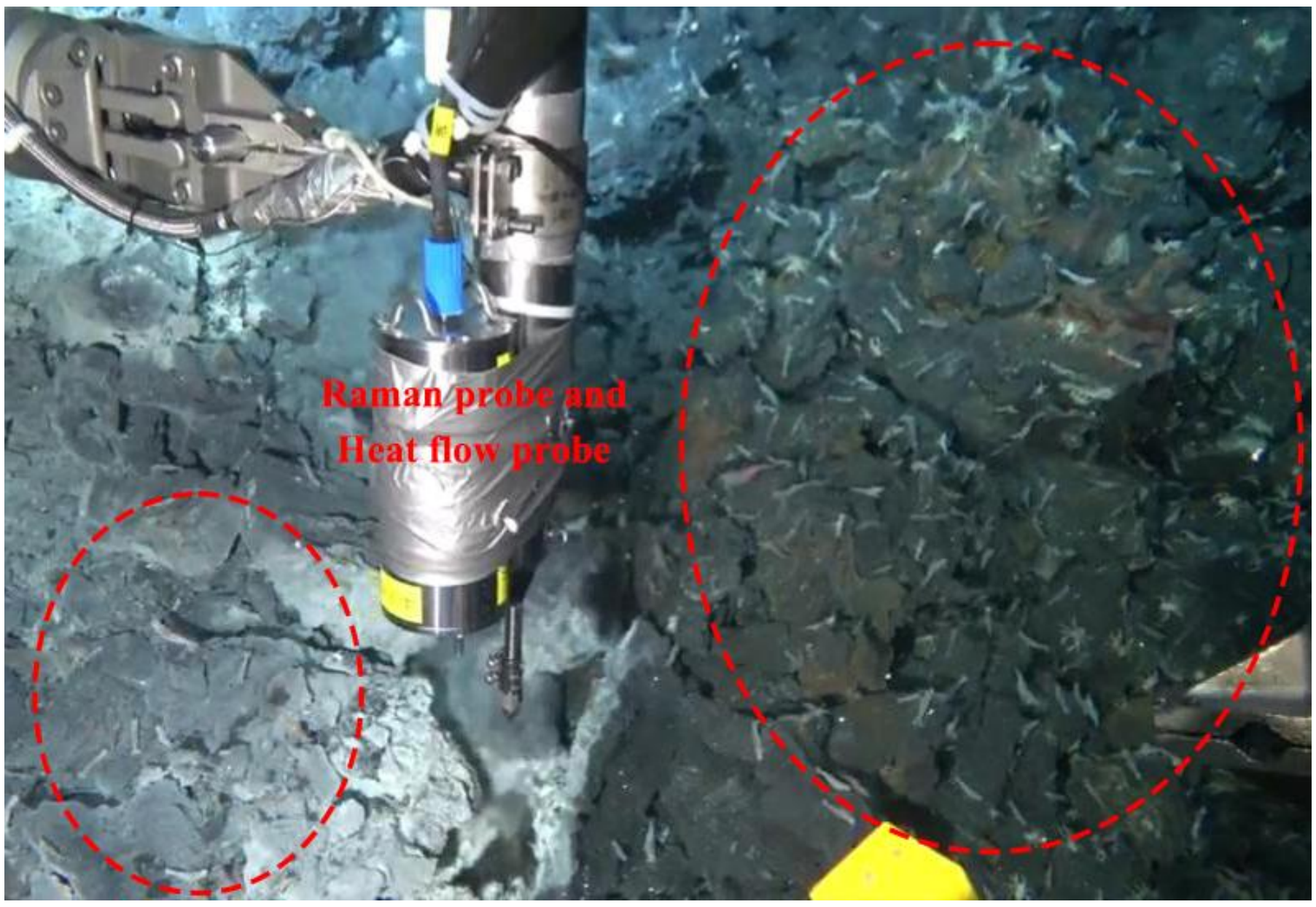

Figure S3 Hydrothermal organisms 2. The photo of the deep-sea in-situ Raman probe working in the deep-sea hydrothermal vent area, surrounded by a large number of Rimicaris exoculata (the photo was taken by the "*Faxian*" ROV in June 2022, in the hydrothermal area of Okinawa, ~2000 m depth).

The dominant populations include *Shinkaia crosnieri* and *Rimicaris exoculata*. In the on-site photos shown in Figures 1b–e and S2 and S3, it can be observed that there were many *S. crosnieri* and *R. exoculata* individuals in the deep-sea hydrothermal zone at a depth of over 2,000 m. Moreover, in Figure 1f–i, a large number of mussels and *S. crosnieri* populations can be seen distributed around the cold seep vents.

First, an approximately 100 nm thick layer of Ag was deposited on carbon fibre using EBD technology. Subsequently, the Aus NP-loaded Ag@CFC substrate was obtained through Au NPs loading and *in situ* growth (i.e. D-SERS sensor core component: Au/Ag@CFC substrate).

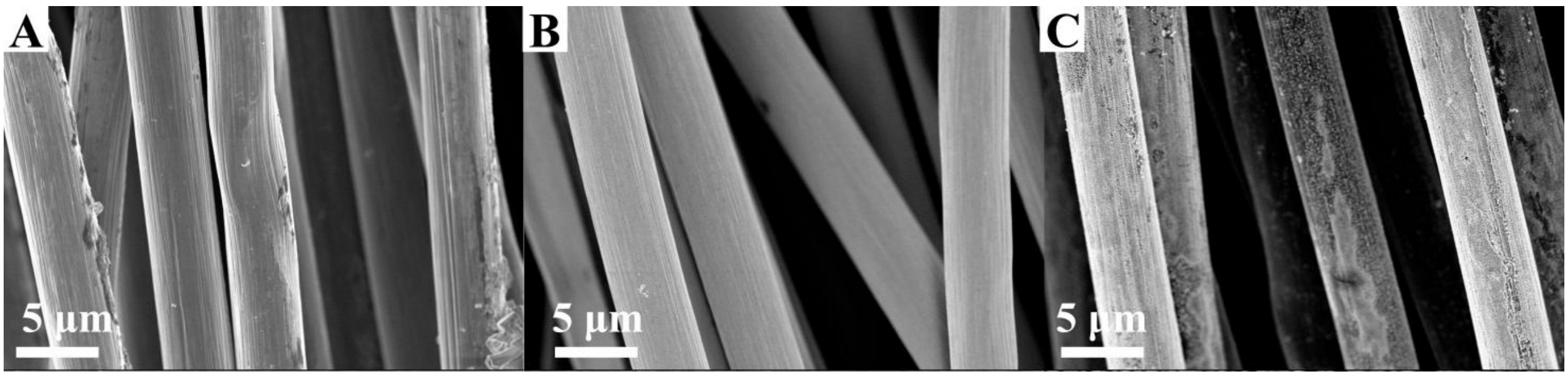

Figure S4 Sensors characterization. The SEM images of (A) CFC, (B) Ag@CFC, and (C) Au/Ag@CFC

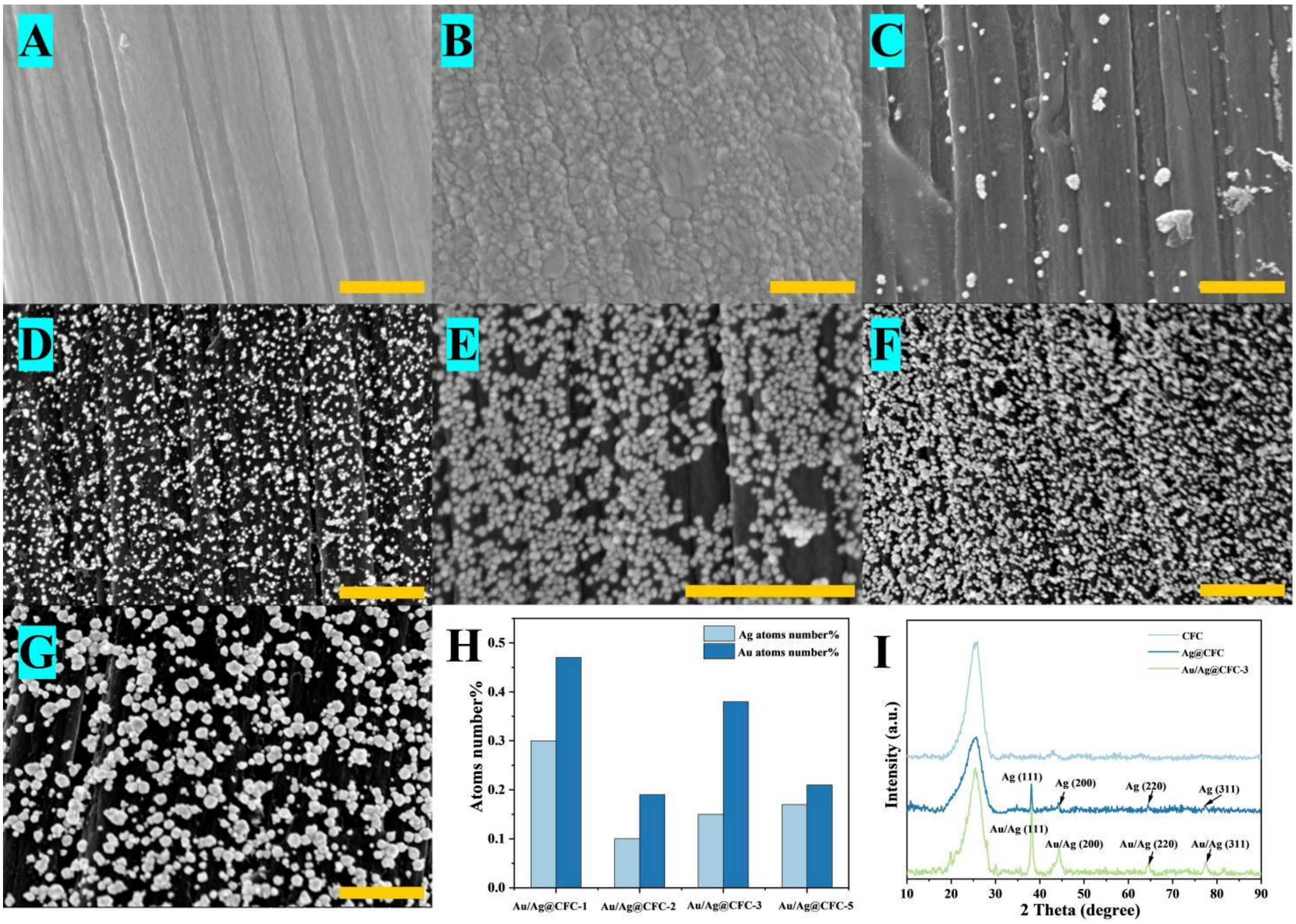

Figure S5 Sensors detailed characterization. Scanning electron microscope images of (A) carbon fibre cloth (CFC), (B) Ag@CFC (C) Au/Ag@CFC-0.5, (D) Au/Ag@CFC-1, (E) Au/Ag@CFC-2, (F) Au/Ag@CFC-3, and (G) Au/Ag@CFC-5 (all scale bars represent 500 nm); (H) bar chart showing the counts of Au and Ag atoms in Au/Ag@CFC-1 to Au/Ag@CFC-5 substrates. (I) X-ray diffraction (XRD) patterns of CFC, Ag@CFC and Au/Ag@CFC-3.

To further demonstrate the successful loading of Ag layers and Au NPs, X-ray diffraction (XRD) analyses were performed on CFC, Ag@CFC and Au/Ag@CFC substrates. Figure S5I shows the XRD patterns of the three substrates, with the graphite carbon peak (002) of the CFC located near 26.6° *(59)*. Characteristic XRD peaks of Ag were formed on the Ag@CFC substrate, with 2θ values corresponding to 38.1° (111), 44.3° (200), 64.5° (220), and 77.4° (311), which confirmed the successful deposition of the Ag layer *(60)*. However, the lattice parameter a values of Au and Ag are extremely close, being 4.078 and 4.086 Å, respectively *(61)*. Therefore, the same 2θ values of approximately 38.1° (111), 44.3° (200), 64.5° (220), and 77.4° (311) can be observed on the Au/Ag@CFC substrate *(62, 63)*. However, a comparative analysis of the XRD characteristic peaks of the Ag@CFC substrate revealed that the characteristic peaks corresponding to Au/Ag on the Au/Ag@CFC substrate were sharper. This phenomenon can be used for a preliminary determination of the loading of Au NPs. In conclusion, XRD and XPS tests can further prove the successful loading of Au/Ag on CFC. The successful preparation of the D-SERS sensor has been preliminarily confirmed.

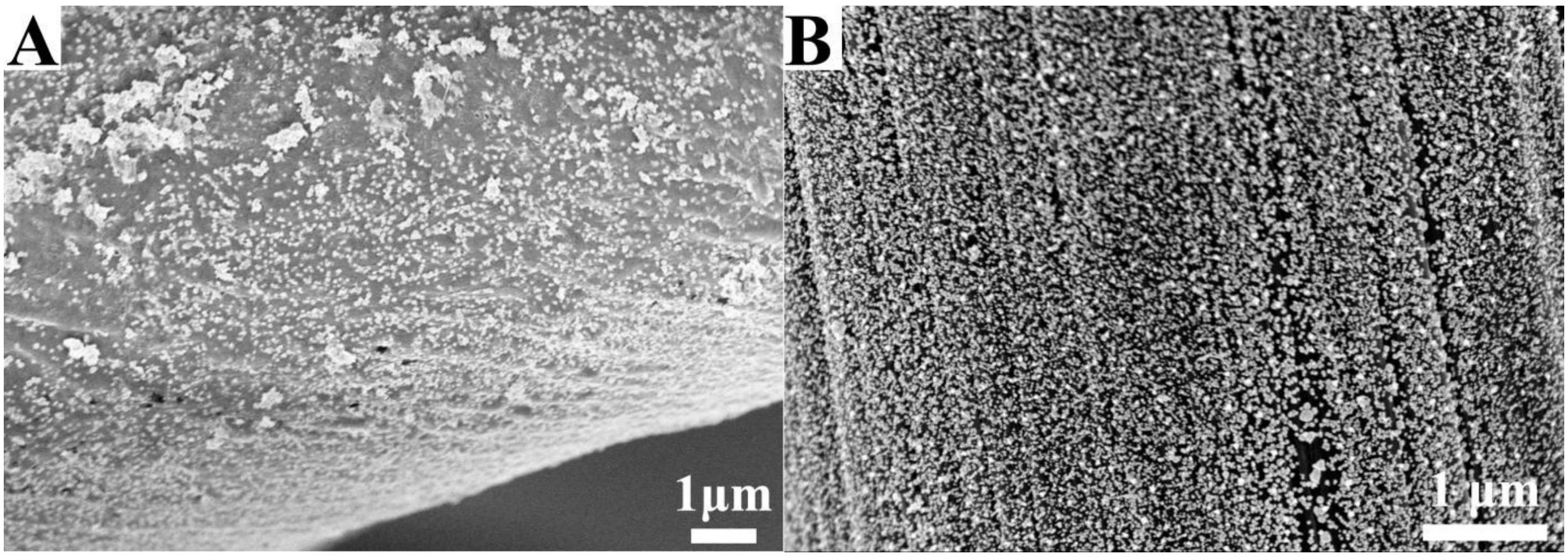

Figure S6 Comparison of SEM images of different sensors. The SEM images of (A) Au@Cloth, (B) Au/Ag@CFC.

Calculation of EF

To measure the degree of Raman signal enhancement of 4-Mpy, the following formula is used:

$EF = [(I_{SERS})/(I_{Normal})] \times [(N_{Normal})/(N_{SERS})]$ (1).

Among them, I_{SERS} obtained the intensity of the 4-Mpy band at 1010 cm^{-1} from the SERS spectrum, I_{Normal} was the peak intensity of the normal Raman spectrum, and N_{SERS} and N_{Normal} were the number of molecules absorbed on the surface of the sample exposed by laser in SERS and normal Raman measurements, respectively (the acquisition conditions of the Raman spectrum were the same). The Raman spectrum of a 0.1 M 4-Mpy solution on a quartz substrate is shown in Figure 3G. Then, N_{SERS} can be calculated as $N_{SERS} = S/S_{4\text{-}Mpy}$, where S and $S_{4\text{-}Mpy}$ are the area of the sample irradiated by the laser and the occupied area of the 4-Mpy molecule, respectively. $N_{Normal} = m/M = (S_{spot} \cdot L \cdot \rho \cdot NA)/M_{4\text{-}Mpy}$, where m and M are the excited 4-Mpy powder weight and 4-Mpy molecular weight, respectively. L is the laser penetration depth of 4-Mpy, ρ is the density of 4-Mpy, S_{spot} is the laser spot area with a radius of 0.9 µm, NA is Avogadro's number, and $M_{4\text{-}Mpy}$ is the molar mass of 4-Mpy.

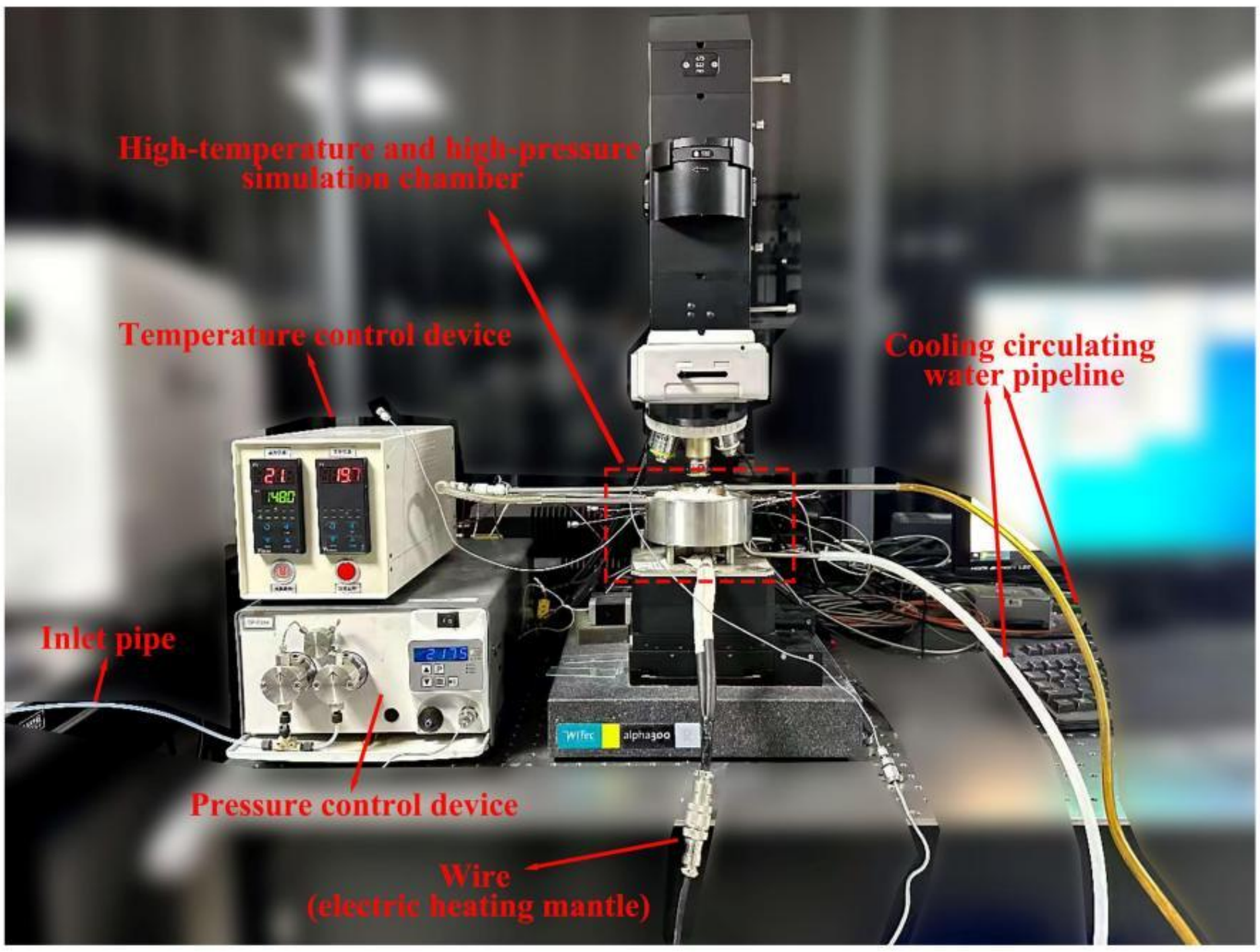

Figure S7 Test system. Photo of the high-temperature and high-pressure simulation device during its working.

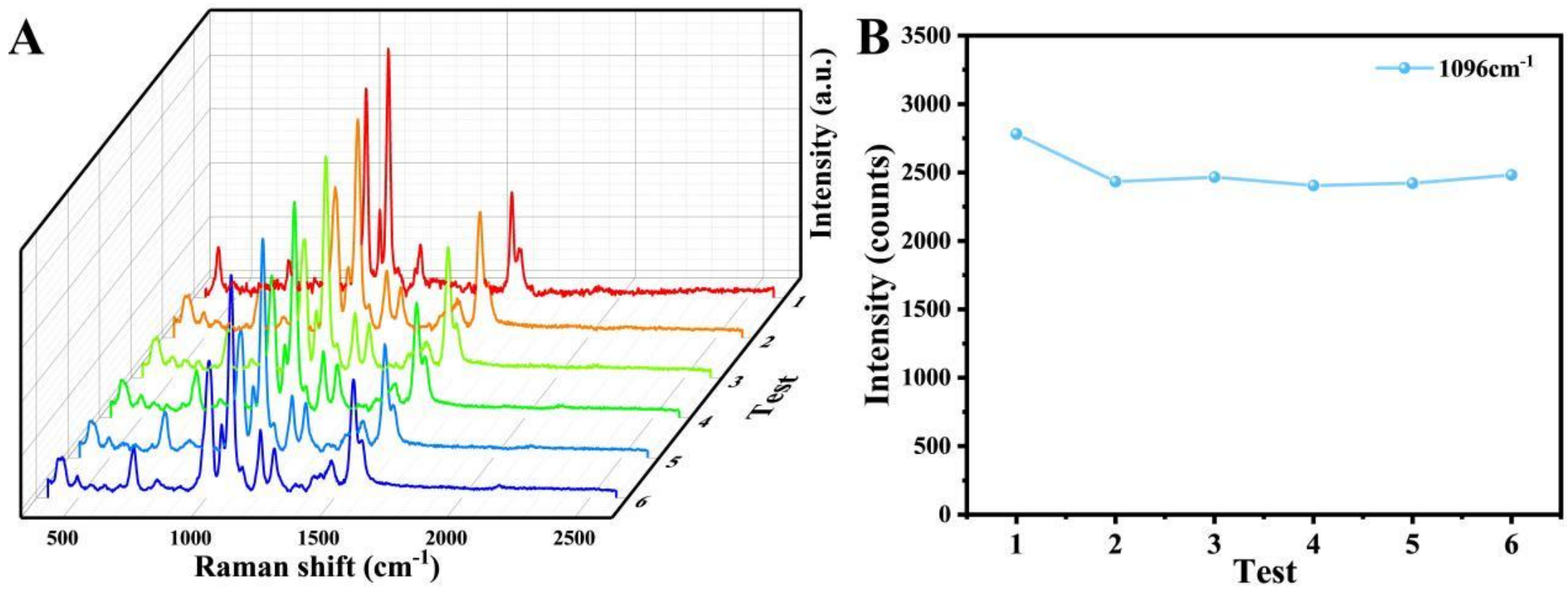

Figure S8 Stability test of D-SERS sensor. (A) Curve 1 represents the 4-Mpy (10^{-5} M) spectrum obtained by the D-SERS sensor in the performance characterization. Curves 2 to 6 are the 4-Mpy (10^{-5} M) spectra obtained by the sealed D-SERS sensor (with preservation time ranging from 1 to 2 months). (B) The line graph showing the change of the characteristic peak at 1096 cm^{-1}.

As shown in Figure S8, the spectral profiles (peak positions and relative intensities) of curves 2 to 6 maintain a high degree of consistency with curve 1. This indicates that despite a prolonged storage period, the sensor retains good structural integrity and detection sensitivity. Although there are slight variations in peak intensity, the overall levels are comparable (degradation rate<7.5%), with no abrupt decline observed. This demonstrates the effectiveness of the “sandwich tape sealing” strategy, which successfully prevents the oxidation-induced deactivation or contamination of the sensor’s active sites (such as nanomaterials), ensuring its long-term reliable application in harsh environments like cold seeps. Figure S8 strongly validates the excellent long-term stability of the developed D-SERS sensor. Even after 1-2 months of sealed storage, the sensor’s performance in detecting 4-Mpy (including both spectral shape and characteristic peak intensity) shows almost no degradation. This provides robust data support for the sensor’s deployment in practical in-situ detection in cold seeps.

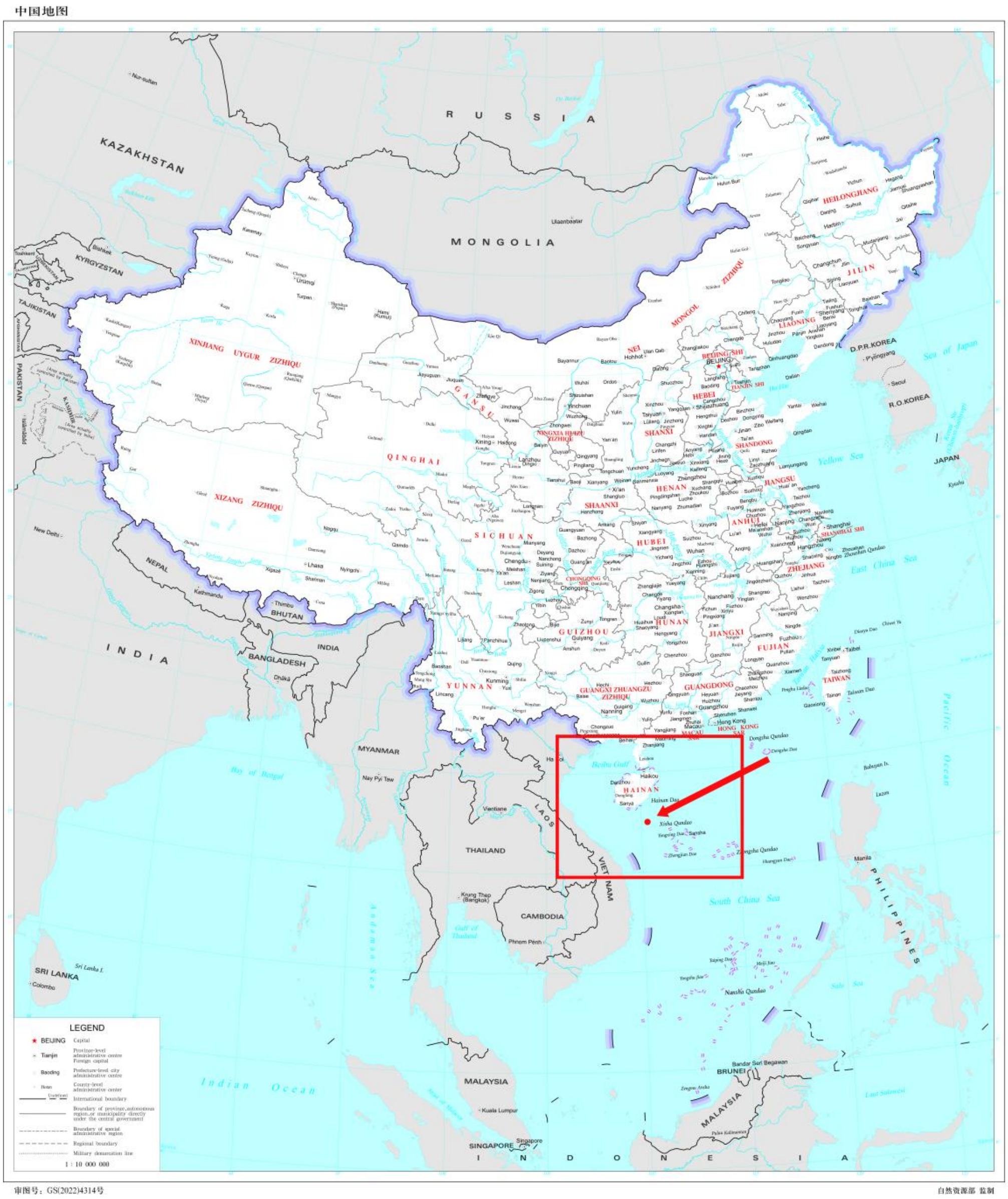

Figure S9 The site of *Hamai* cold seep. The base map of this image is sourced from a free public website - Standard Map Service (http://bzdt.ch.mnr.gov.cn/). And it was marked with Photoshop 2024.

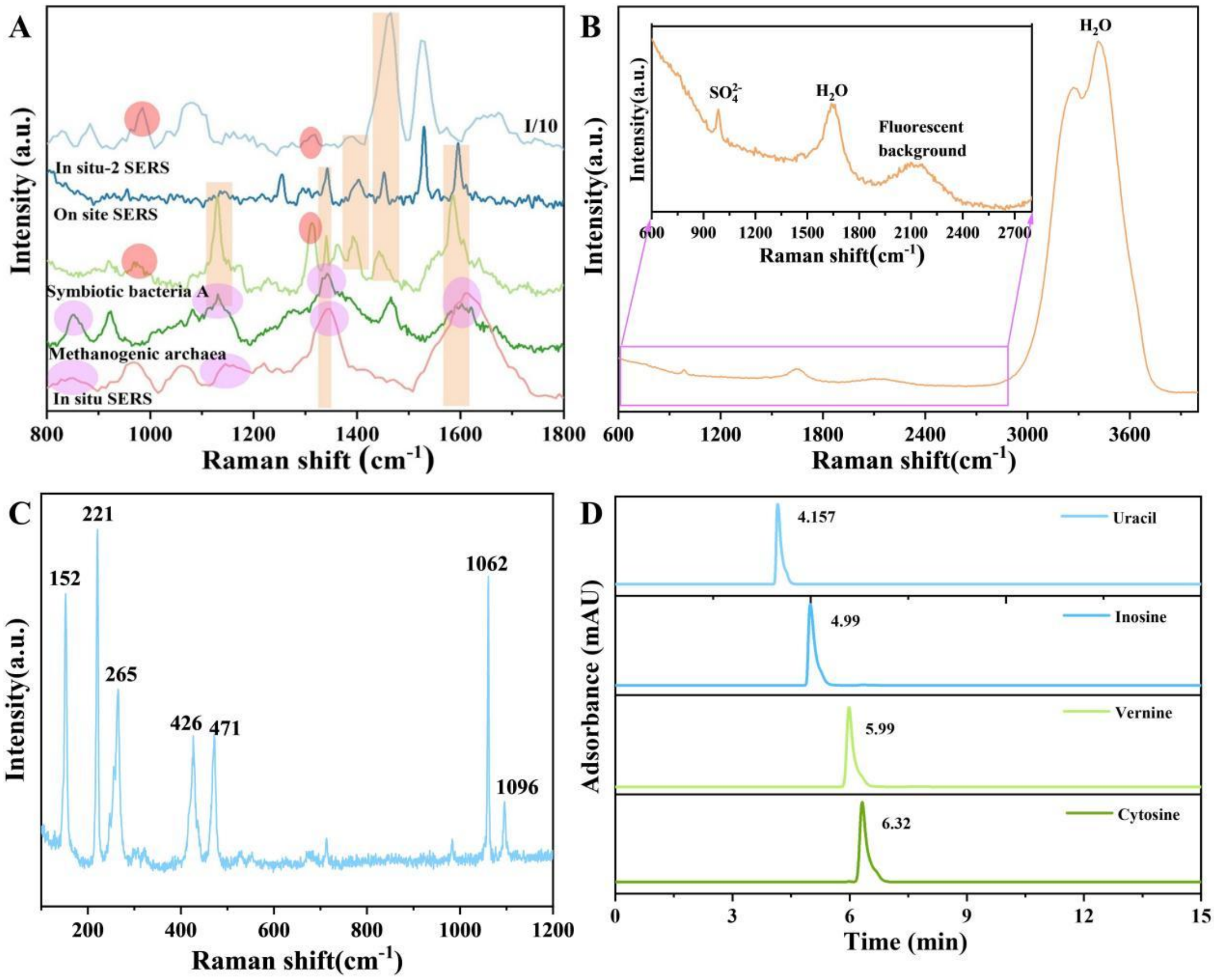

Figure S10 Based on supplementary data from in-situ experiments. (A) on-site test data, *in situ,* and *in situ*-2 deep-sea test data, and several Raman spectra of microorganisms from the deep sea are compared; (B) The ordinary Raman spectrogram obtained from deep-sea in-situ collection (obtained in the *in situ*-2 area); (C) The Raman spectrum diagram of the standard substance of tetra sulfate. (D) liquid chromatograms of four nucleotide (base) standards (uracil, inosine, guanosine and cytosine).

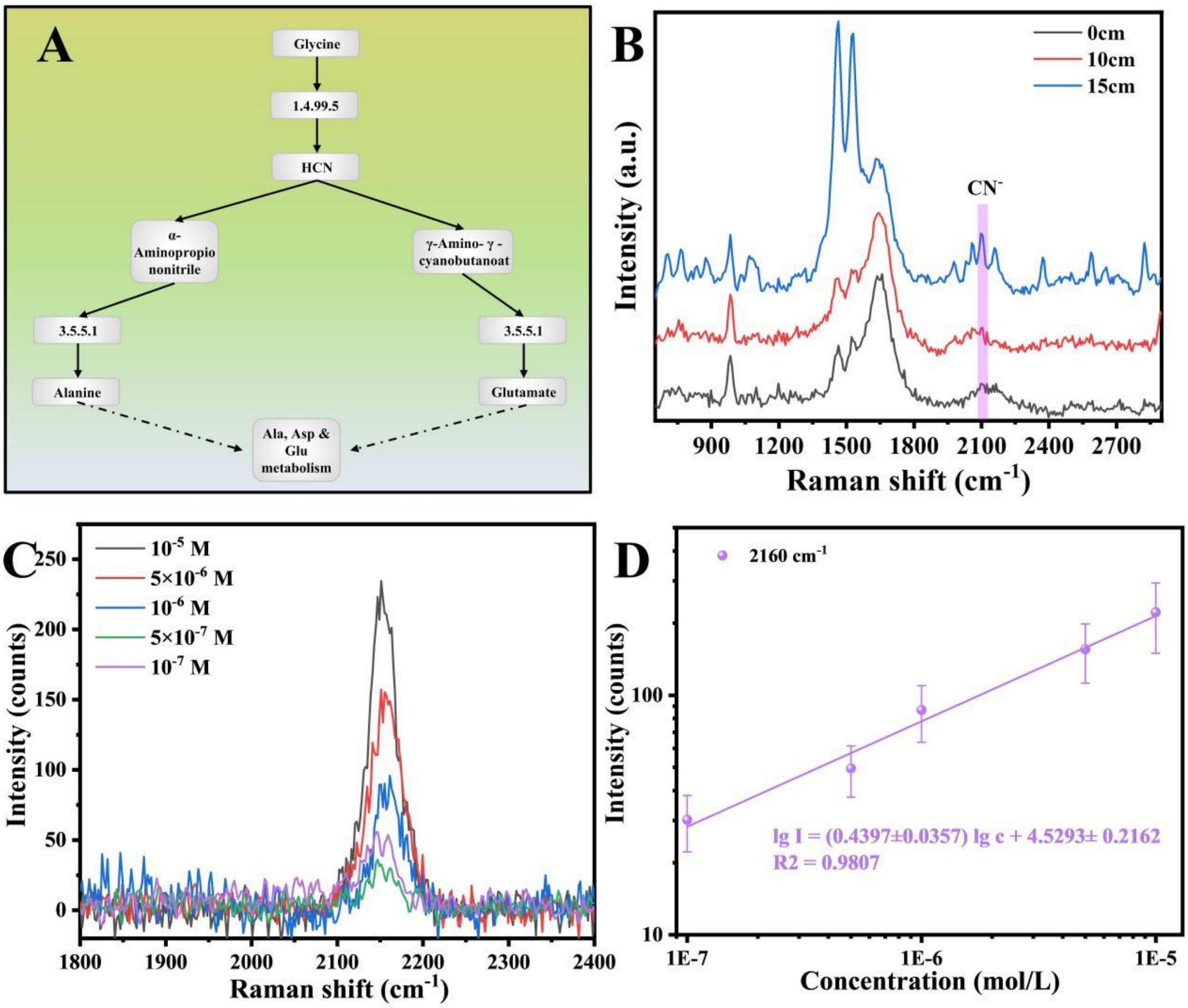

Figure S11 Cyanide test. (A) Based on the KEGG pathway: Cyanoamino acid metabolism - *Pseudomonas*; (B)The SERS detection spectral data obtained through gradient detection above the *in situ*-2 area, with the distances to the biological community being 0, 10 and 15 cm respectively; (C) SERS spectra of different concentrations of -*CN* in the range of 10^{-5} to 10^{-7} M with the same soaking time. (D) Intensities of the Raman characteristic peaks of -*CN* at 2160 cm^{-1} varying from 10^{-5} to 10^{-7} M (logarithmic scale) with varying molecular concentrations (logarithmic scale) corresponding to those shown in (C).

The quantitative model of -*CN* was obtained using potassium ferrocyanide as the model. The specific steps are as follows: First, prepare solutions of different concentrations of degradation rate (ranging from 10^{-5} to 10^{-7} M). Then, immersed the SERS substrate in the solution for 20 min, dried it, and washed it with deionized water. Finally, a Raman test was conducted to obtain the Raman spectra of -*CN* at different concentrations.

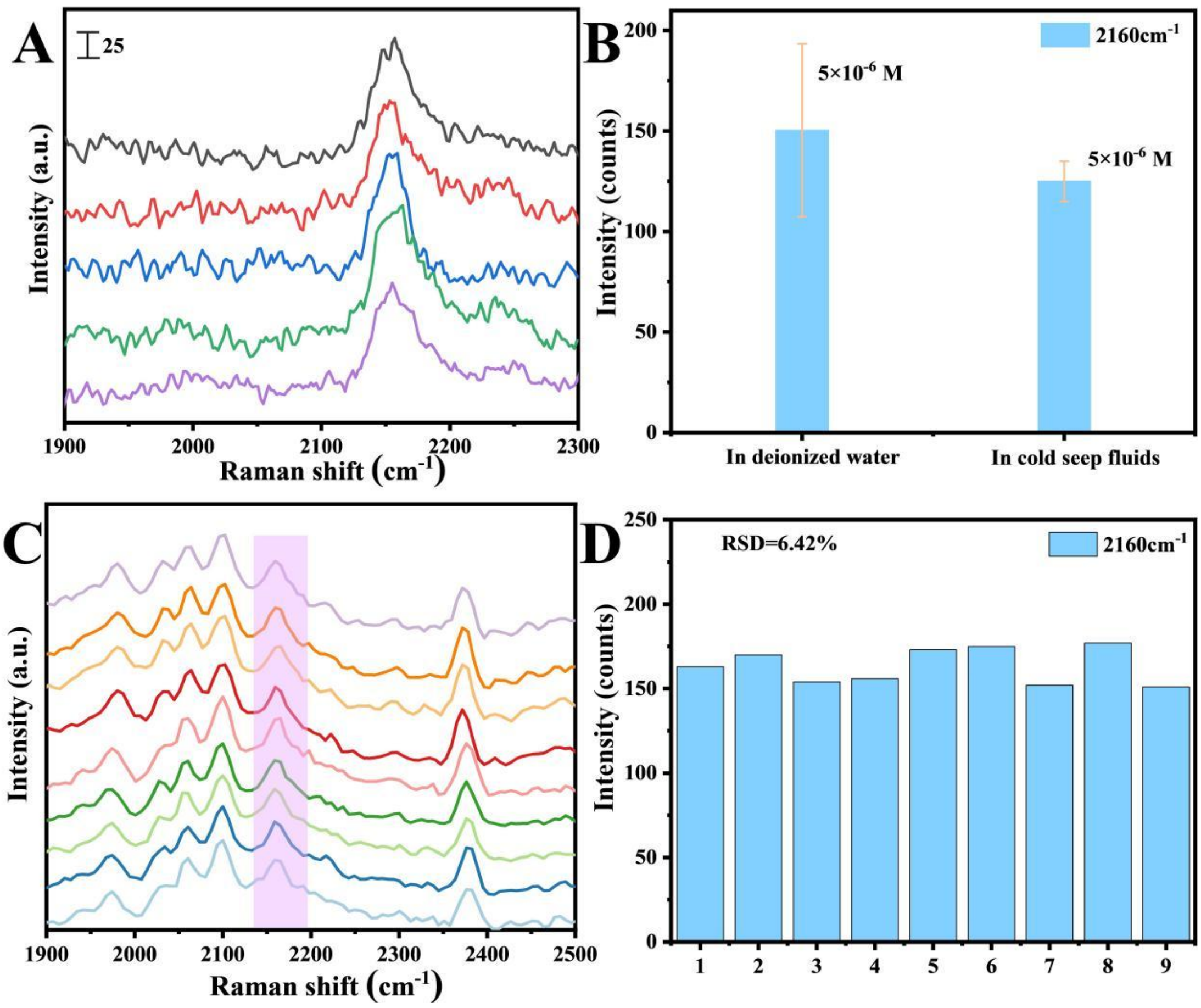

Figure S12 Stability tests of D-SERS Sensors in the deep sea. (A) Using the cold spring pure fluid as the solvent, prepare a potassium ferricyanide solution with a final concentration of 5×10^{-6} M, and obtain the spectral data from the D-SERS sensor; (B) Comparison of Raman signals in deionized water solution and cold spring fluid solution (at 2160 cm^{-1}); (C) Comparison of Raman signals collected at in situ-2; (D) Bar chart of the signal peak intensity at 2160 cm^{-1} and RSD analysis.

At each of our in-situ sites, we employed two D-SERS sensors for technical replication. Each D-SERS sensor conducted 4-5 spectral acquisitions to evaluate the repeatability of the in-situ measurements. We also supplemented the statistical analysis of the intensity of the cyanide characteristic peak (2160 cm^{-1}). Specifically, in the in-situ measurement at in situ-2, from the 9 spectra obtained by the two sensors in repetition, the relative standard deviation (RSD) of the Raman peak intensity at 2160 cm^{-1} (C≡N stretching vibration) was 6.42% (Figure S12C-D). This demonstrates the excellent reproducibility of the D-SERS sensors in detecting this key biogeochemical marker in the extreme deep-sea environment.

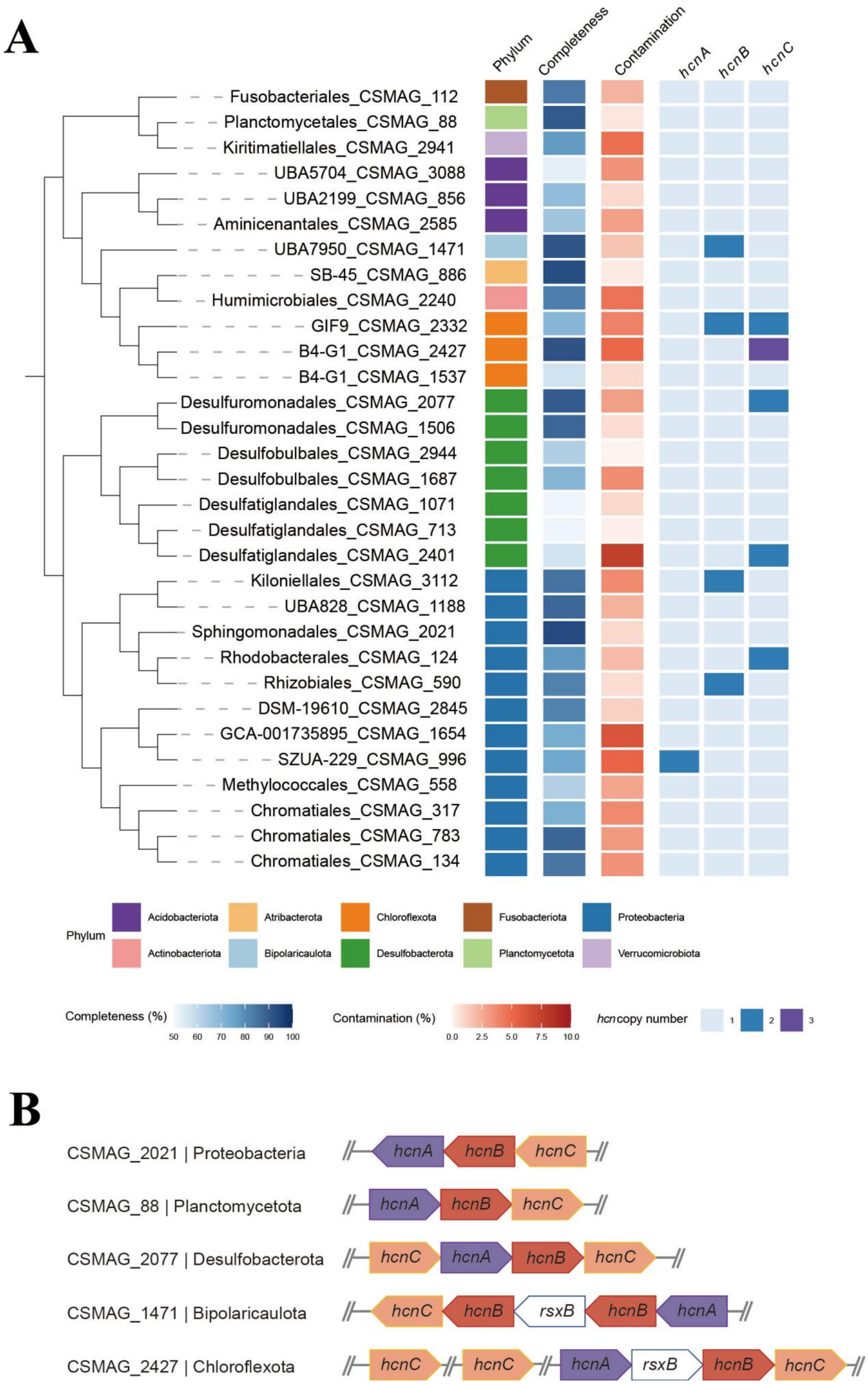

A
Phylum
Completeness
Contamination
hcnA
hcnB
hcnC
Fusobacteriales_CSMAG_112
Planctomycetales_CSMAG_88
Kiritimatiellales_CSMAG_2941
UBA5704_CSMAG_3088
UBA2199_CSMAG_856
Aminicenantales_CSMAG_2585
UBA7950_CSMAG_1471
SB-45_CSMAG_886
Humimicrobiales_CSMAG_2240
GIF9_CSMAG_2332
B4-G1_CSMAG_2427
B4-G1_CSMAG_1537
Desulfuromonadales_CSMAG_2077
Desulfuromonadales_CSMAG_1506
Desulfobulbales_CSMAG_2944
Desulfobulbales_CSMAG_1687
Desulfatiglandales_CSMAG_1071
Desulfatiglandales_CSMAG_713
Desulfatiglandales_CSMAG_2401
Kiloniellales_CSMAG_3112
UBA828_CSMAG_1188
Sphingomonadales_CSMAG_2021
Rhodobacterales_CSMAG_124
Rhizobiales_CSMAG_590
DSM-19610_CSMAG_2845
GCA-001735895_CSMAG_1654
SZUA-229_CSMAG_996
Methylococcales_CSMAG_558
Chromatiales_CSMAG_317
Chromatiales_CSMAG_783
Chromatiales_CSMAG_134
Phylum
Acidobacteriota
Atribacterota
Chloroflexota
Fusobacteriota
Proteobacteria
Actinobacteriota
Bipolaricaulota
Desulfobacterota
Planctomycetota
Verrucomicrobiota
Completeness (%)
50 60 70 80 90 100
Contamination (%)
0.0 2.5 5.0 7.5 10.0
hcn copy number
1 2 3
B
CSMAG_2021 | Proteobacteria
hcnA hcnB hcnC
CSMAG_88 | Planctomycetota
hcnA hcnB hcnC
CSMAG_2077 | Desulfobacterota
hcnC hcnA hcnB hcnC
CSMAG_1471 | Bipolaricaulota
hcnC hcnB rsxB hcnB hcnA
CSMAG_2427 | Chloroflexota
hcnC hcnC hcnA rsxB hcnB hcnC

Figure S13. Phylogenetic distribution and genomic organization of hcnABC-containing MAGs.

(A) Genome tree of 31 cold seep MAGs carrying putative hcn genes. The tree was generated using GTDB-Tk based on the bacterial 120 conserved marker genes. The tip labels indicate taxonomic order-level assignment followed by MAG ID. The phylum-level taxonomy, genome completeness, and contamination of these MAGs were obtained from Han et al. *(54)*. Side annotation tracks show phylum, completeness, contamination, and copy numbers of hcnA, hcnB, and hcnC.

(B) Representative genomic neighborhoods of hcnABC loci in some high-quality MAGs. Genes were predicted using Prokka and annotated using Pfam. Arrows indicate gene orientation.

Supplementary Text S2:

Phylogenetic distribution and genomic organization of hcnABC-containing MAGs

The hcnABC gene cluster encodes hydrogen cyanide synthase, a membrane-associated enzyme complex responsible for bacterial HCN biosynthesis. To assess the distribution of potential HCN-producing microorganisms in cold seep ecosystems, we screened the 3,164 species-level MAGs from the global cold seep genomic catalog. A total of 407 MAGs possessed at least one subunit of the hcn gene cluster, and 31 MAGs contained all three subunits. These MAGs spanned multiple bacterial phyla, including Proteobacteria, Desulfobacterota, Chloroflexota, Planctomycetota, Acidobacteriota, Verrucomicrobiota, Bipolaricaulota, Actinobacteriota, Atribacterota, and Fusobacteriota (Fig. S13A). In addition, the copy numbers of hcnA, hcnB, and hcnC differed across MAGs, suggesting that the genetic potential for hydrogen cyanide-related metabolism is broadly distributed among taxonomically distinct microbial groups in fcold seep ecosystems.

Among these MAGs, CSMAG_588, classified as a WTBX01 genus member within Methylococcales, was one of the dominant populations in seepage-influenced waters and was particularly enriched at the bottom water interface. As WTBX01-related Methylococcales harbor key genes involved in methane oxidation and downstream C1 metabolism, this MAG likely participates in methane oxidation and carbon turnover at the seep–water interface, while also possessing the potential for HCN biosynthesis *(60)*.

Representative genomic neighborhoods further revealed substantial structural variation in hcn loci (Fig. S13B). Some MAGs, such as CSMAG_2021 and CSMAG_88, contained compact and co-oriented hcnA-hcnB-hcnC clusters, whereas others showed additional gene copies or rearranged architectures. For example, CSMAG_2077 contained an extra hcnC copy, while CSMAG_1471 and CSMAG_2427 harbored rsxB (Ion-translocating oxidoreductase complex subunit B) within or adjacent to the hcn gene neighborhood.

The presence of compact hcnABC clusters in some MAGs indicates that these genes often occur as complete genomic modules, whereas rearranged or duplicated hcn loci in other MAGs suggest structural diversification of hcn-associated genomic regions. Together, these results reveal phylogenetically diverse hcnABC-containing

microorganisms in global cold seep MAGs and suggest that these microorganisms may contribute to cyanide or hydrogen cyanide production in deep-sea cold seep environments.

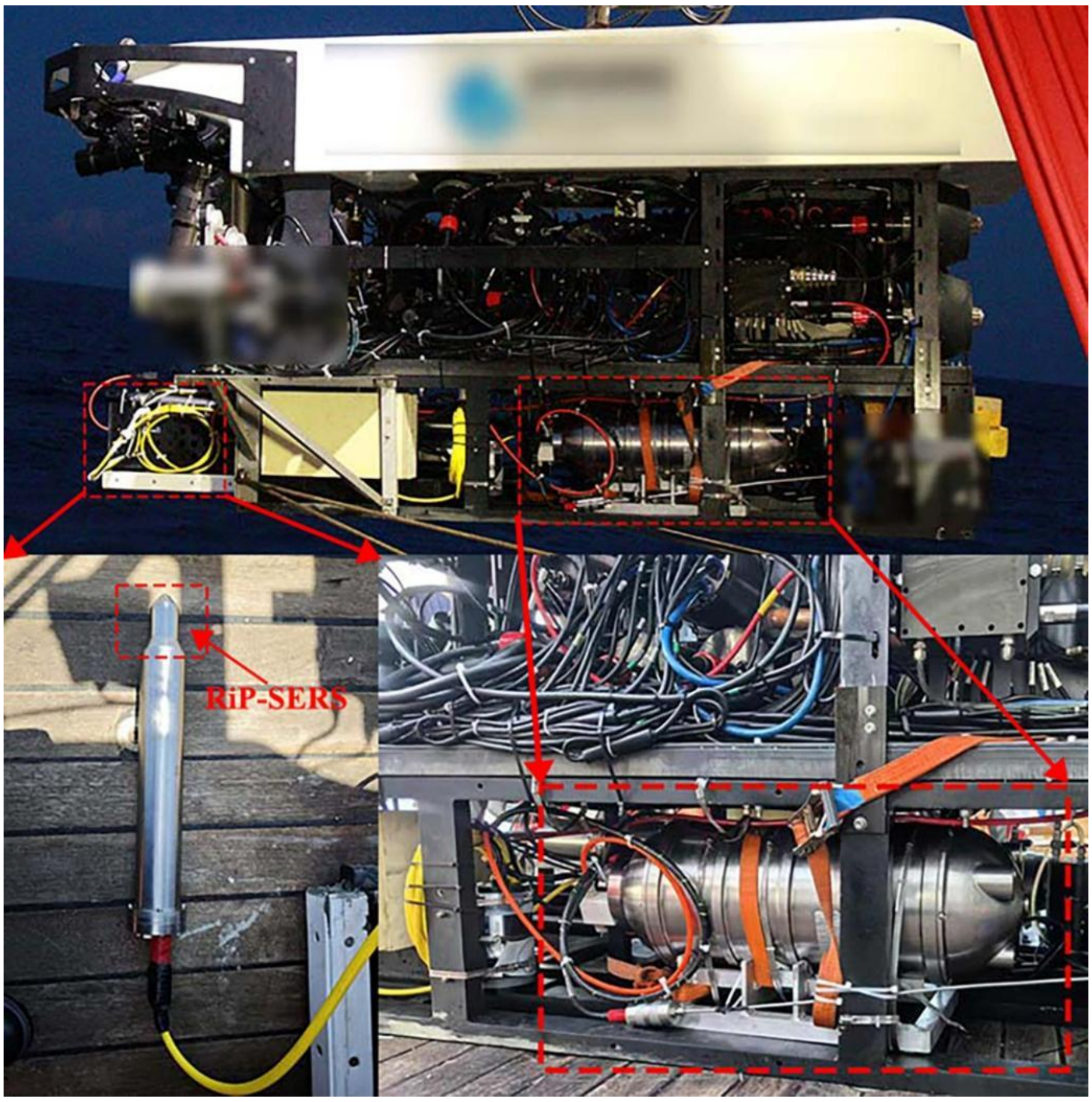

Figure S14 RiP system. Physical photos of the deep-sea in-situ Raman detection system carrying an ROV and detailed enlarged photos of the Raman system (taken on May, 2025, on the *Deep-sea No.1* scientific research vessel).

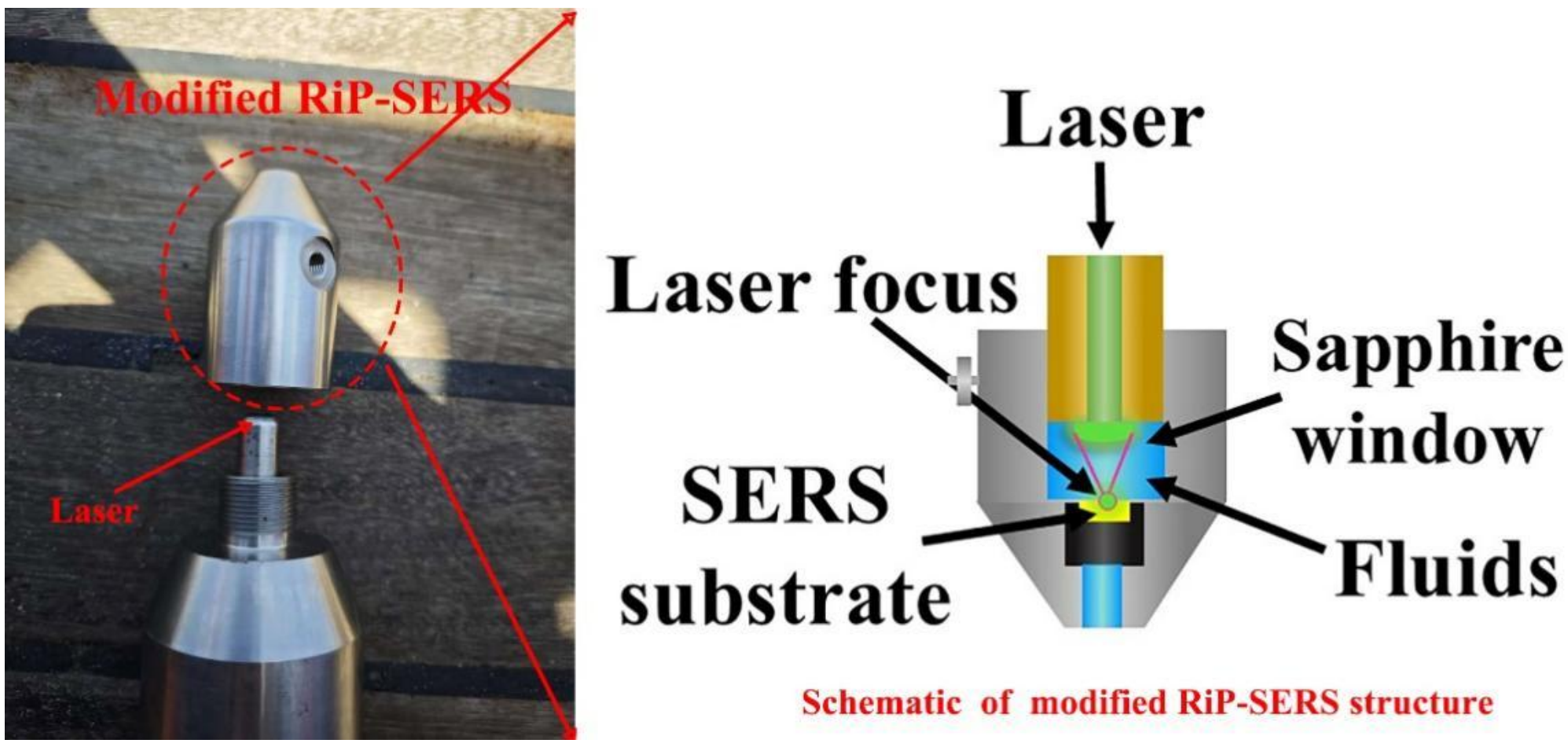

Figure S15 RiP-SERS probe. The left picture shows the photos of the modified RiP-SERS probe and the Raman laser window used in the on-site test, and the right picture shows the structural schematic diagram of the modified RiP-SERS probe (Wang et al. *(18)*).

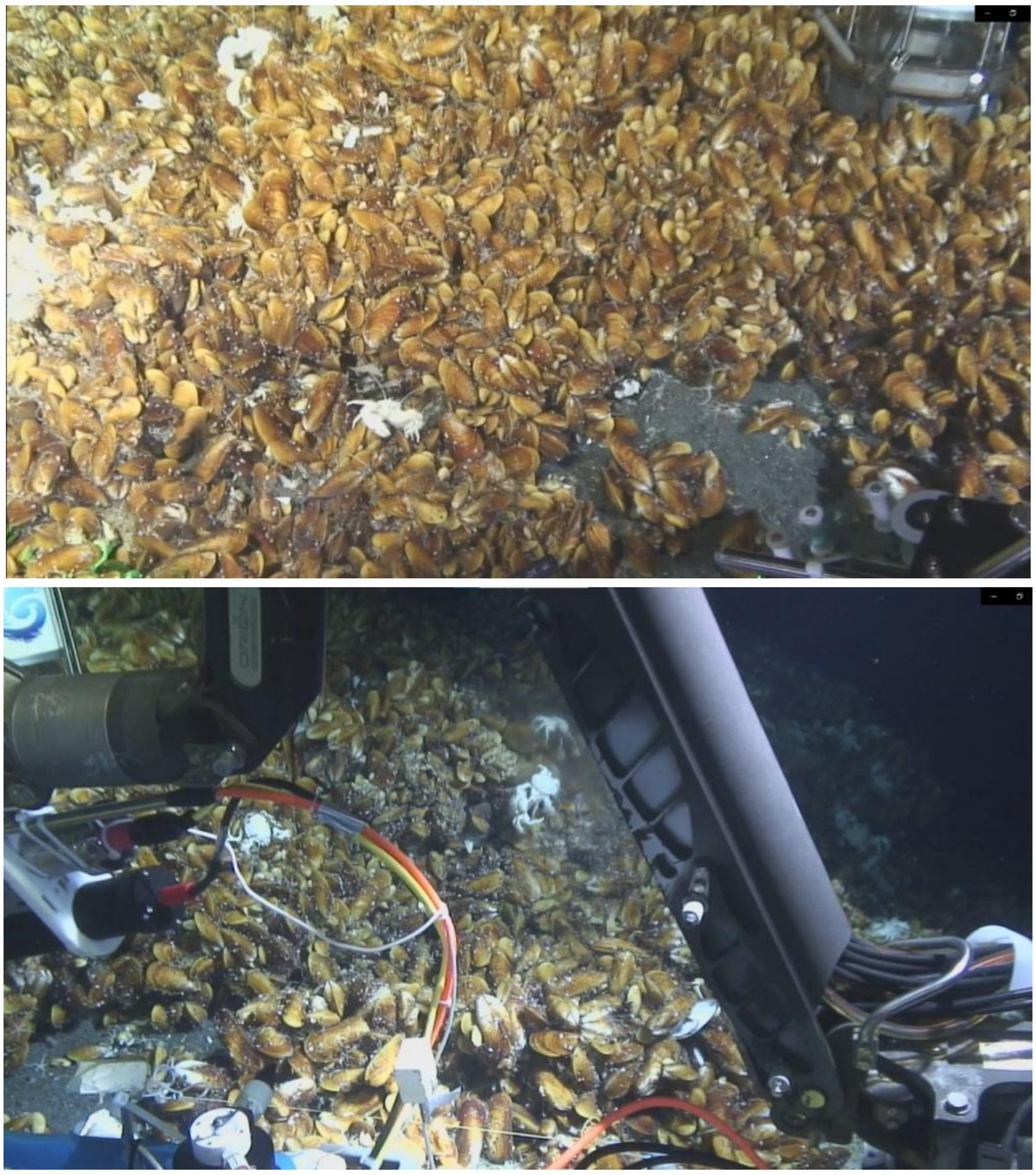

Figure S16 Photos of in-situ work in the deep sea. The photos of the biological community in the *Haima* Cold Seep Area (taken in May 2025, aboard the HOV of *Jiaolong*).

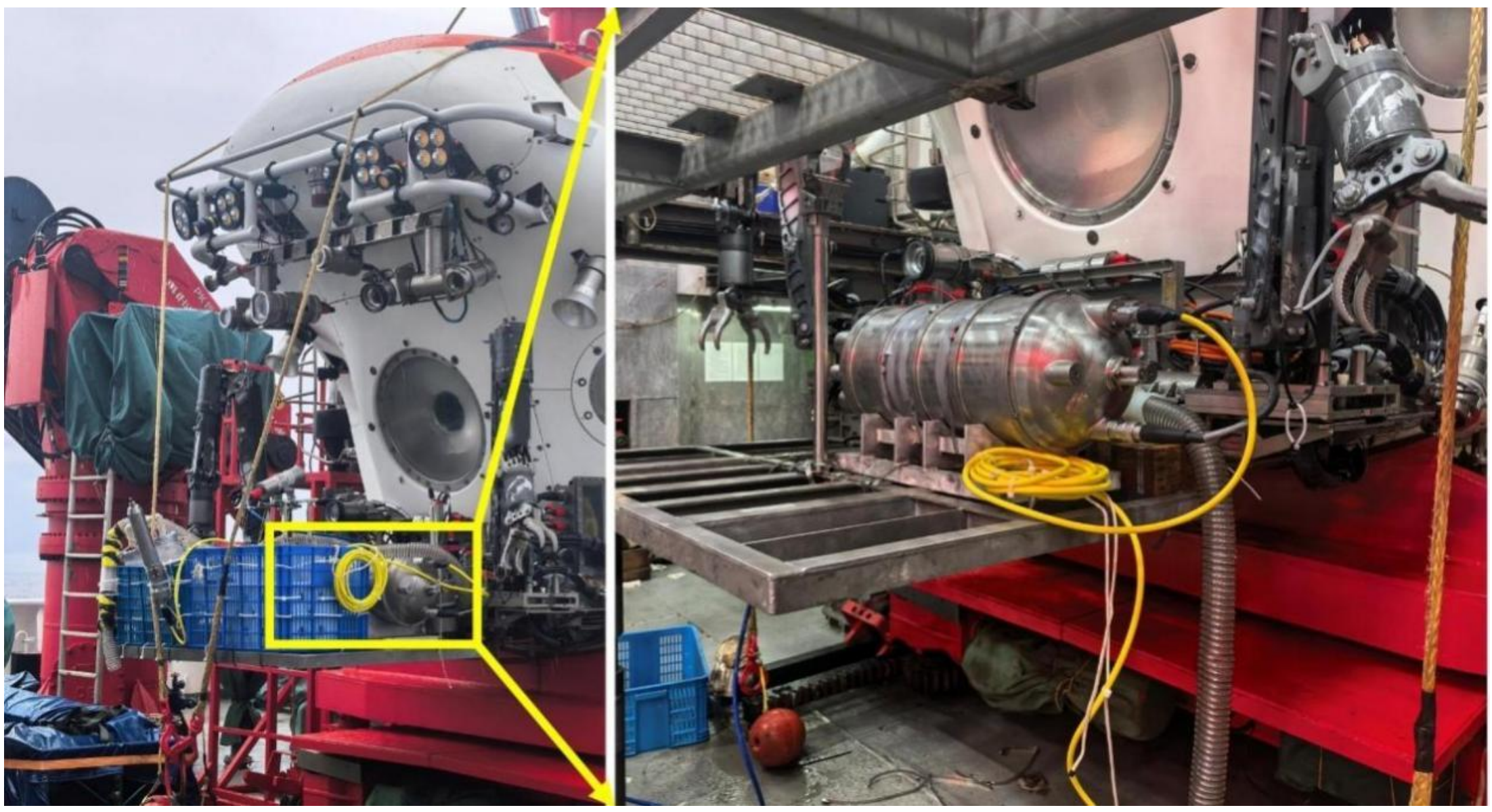

Figure S17 The RiP system is equipped with photos. A photo of the RiP system

mounted on the front loading frame of the "*Jiaolong*" manned submersible (the photo was taken in May 2025)

Table S1 The functional groups corresponding to the Raman shifts within the range of 600-1800 cm^{-1} obtained at the position of the *in situ* site and the associated biomarkers.

Raman shift/cm^{-1}	Vibrational Assignment	Biological Assignment	Reference
747	Indole ring bending	Tryptophan	*(40)*
851	1,4-glycosidic link stretching	Polysaccharide structure	
951	CH_3 symmetric stretching	Proteins	
1065	Skeletal C-C stretching or	Lipids	
1199	Ring breathing	Tyrosine	
1239	C–C–H in-plane rocks	Bacteriorhodopsin	
1609	Phenyl ring bond-stretching vibrations(in-phase)	Phenylalanine	*(39)*

Table S2 The functional groups corresponding to the Raman shifts within the range of 600–1800 cm^{-1} obtained at the position of the *in situ*-2 site and the associated biomarkers

Raman shift/cm^{-1}	Vibrational Assignment	Biological Assignment	Reference
771	Ring breathing	Uracil	*(40)*
875	out-of-plane bending of indole ring and indole CH	Tryptophan	
984	SO_4^{2-} symmetrical stretching (ν_1)	SO_4^{2-}	*(10)*
1064	Skeletal C-C stretching or	Lipids or	Figure

	S-O	polythionate	S11b and *(58, 64)*
1095	PO_2^- symmetric stretching or SO_4^{2-}	DNA or Thionate	
1160	C-C stretching	Carotenoid	*(65)*
1313	C-H deformation	Carotenoid	
1465	CH_2 bending	Proteins and lipids	*(40)*
1528	C=C stretching	Carotenoid	*(65)*
1660	C-O stretching vibrations out-of-plane C-N stretching	Amide I	*(40)*

Table S3 Comparison of the advantages between D-SERS sensors and RiP-SERS sensors *(18)*.

Feature	This work: D-SERS Sensor	Wang et al., 2023 *(18)* (RiP-SERS)
Detection Principle	SERS	SERS
Sensitivity (LOD)	nM level	nM level
Pressure Tolerance	> 22 MPa (2200 m)	> 11 MPa (1100 m)
Temperature Range	2 - 350 °C (tested)	2 - 30 °C (tested)
Quantity per deployment	> 100 pieces	1 piece
Points per measurement	Multiple-point detection	Single-point detection

Movie S1.
At Site 1 of the *Haima* cold seep in the South China Sea, after descending to ~1,500 m aboard the HOV Jiaolong, we performed in-situ SERS detection with a D-SERS sensor. The video footage shows sparse organisms, indicating the cold-seep community is in a declining phase.

Movie S2.
At Site 2 of the *Haima* cold seep in the South China Sea, the HOV “Jiaolong”descended to approximately 1,200 m (the depth of the cold seep here differs from Site 1 because the Haima system covers a broad area with highly variable seafloor topography). Once on the bottom, we performed in-situ SERS measurements using a D-SERS sensor. The recorded video shows exceptionally high biomass, indicating that the cold-seep community at this location is in a flourishing stage.

Movie S3.
Video of the D-SERS sensor being replaced *in situ* in the deep sea.

REFERENCES

1. X. Dong, C. Zhang, Y. Peng, H.-X. Zhang, L.-D. Shi, G. Wei, C. R. J. Hubert, Y. Wang, C. Greening, Phylogenetically and catabolically diverse diazotrophs reside in deep-sea cold seep sediments. *Nat. Commun.* **13**, 4885 (2022).

2. R. W. Fulweiler, Fantastic Fixers. *Science* **326**, 377–378 (2009).

3. A. E. Dekas, R. S. Poretsky, V. J. Orphan, Deep-sea archaea fix and share nitrogen in methane-consuming microbial consortia. *Science* **326**, 422–426 (2009).

4. Q. Jiang, L. Cao, Y. Han, S. Li, R. Zhao, X. Zhang, S. E. Ruff, Z. Zhao, J. Peng, J. Liao, B. Zhu, M. Wang, X. Lin, X. Dong, Cold seeps are potential hotspots of deep-sea nitrogen loss driven by microorganisms across 21 phyla. *Nat. Commun.* **16**, 1646 (2025).

5. Q.-L. Sun, K. Xu, L. Cao, Z. Du, M. Wang, L. Sun, Nitrogen and sulfur cycling driven by Campylobacterota in the sediment–water interface of deep-sea cold seep: A case in the South China Sea. *MBio* **14**, e00117–e00123 (2023).

6. Y. He, H. Zhang, F. Baltar, Y. Wang, Transcriptional difference of deep-sea microorganisms under different sampling methods. *Environ. Sci. Technol.* **59**, 11653–11665 (2025).

7. Q. Quan, J. Liu, X. Xia, S. Zhang, Z. Ke, M. Wang, Y. Tan, Cold seep nitrogen fixation and its potential relationship with sulfur cycling. *Microbiol. Spectr.* **12**, e00536–e00524 (2024).

8. D. K. Ngugi, S. G. Acinas, P. Sánchez, J. M. Gasol, S. Agusti, D. M. Karl, C. M. Duarte, Abiotic selection of microbial genome size in the global ocean. *Nat. Commun.* **14**, 1384 (2023).

9. S. Xi, R. Huang, Z. Luan, Z. Du, L. Li, G. Xie, W. Sun, X. Zhang, Quantitative in situ Raman monitoring of formation of abundant formate during the hydrogenation of carbon dioxide. *Sci Bull* **70**, 500–503 (2025).

10. L. Li, H. Zhang, S. Xi, C. Liu, C. Li, J. Zheng, J. Huang, Y. Hao, X. Zhang, W. Sun, A large intraplate hydrogen-rich hydrothermal system driven by serpentinization in the western Pacific: Kunlun. *Sci. Adv.* **11**, eadx3202 (2025).

11. K. S. Lee, Z. Landry, A. Athar, U. Alcolombri, P. P. N. Ayutthaya, D. Berry, P. de Bettignies, J.-X. Cheng, G. Csucs, L. Cui, V. Deckert, T. Dieing, J. Dionne, O. Doskocil, G. D'Souza, C. García-Timermans, N. Gierlinger, K. Goda, R. Hatzenpichler, R. J. Henshaw, W. E. Huang, I. Iermak, N. P. Ivleva, J. Kneipp, P. Kubryk, K. Küsel, T. K. Lee, S. S. Lee, B. Ma, C. Martínez-Pérez, P. Matousek, R. U. Meckenstock, W. Min, P. Mojzeš, O. Müller, N. Kumar, P. H. Nielsen, I. Notingher, M. Palatinszky, F. C. Pereira, G. Pezzotti, Z. Pilat, F. Plesinger, J. Popp, A. J. Probst, A. Riva, A. A. E. Saleh, O. Samek, H. M. Sapers, O. T. Schubert, A. K. M. Stubbusch, L. F. Tadesse, G. T. Taylor, M. Wagner, J. Wang, H. Yin, Y. Yue, R. Zenobi, J. Zini, U. Sarkans, R. Stocker, MicrobioRaman: An open-access web repository for microbiological Raman spectroscopy data. *Nat. Microbiol.* **9**, 1152–1156 (2024).

12. K. S. Lee, F. C. Pereira, M. Palatinszky, L. Behrendt, U. Alcolombri, D. Berry, M. Wagner, R. Stocker, Optofluidic Raman-activated cell sorting for targeted genome retrieval or cultivation of microbial cells with specific functions. *Nat. Protoc.* **16**, 634–676 (2021).

13. H. Lin, S. Seitz, Y. Tan, J.-B. Lugagne, L. Wang, G. Ding, H. He, T. J. Rauwolf, M. J. Dunlop, J. H. Connor, J. A. Porco, L. Tian, J.-X. Cheng, Label-free nanoscopy of cell metabolism by ultrasensitive reweighted visible stimulated Raman scattering. *Nat. Methods* **22**, 1040–1050 (2025).

14. L. Wei, F. Hu, Y. Shen, Z. Chen, Y. Yu, C.-C. Lin, M. C. Wang, W. Min, Live-cell imaging of alkyne-tagged small biomolecules by stimulated Raman scattering. *Nat. Methods* **11**, 410–412 (2014).

15. S. Wang, S. Xi, R. Pan, Y. Yang, Z. Luan, J. Yan, X. Zhang, One-step method to prepare coccinellaseptempunctate-like silver nanoparticles for high sensitivity SERS detection. *Surf. Interfaces* **35**, 102440 (2022).

16. S. Laing, L. E. Jamieson, K. Faulds, D. Graham, Surface-enhanced Raman spectroscopy for in vivo biosensing. *Nat Revs Chem* **1**, 0060 (2017).

17. X. Bi, D. M. Czajkowsky, Z. Shao, J. Ye, Digital colloid-enhanced Raman spectroscopy by single-molecule counting. *Nature* **628**, 771–775 (2024).

18. S. Wang, R. Pan, W. He, L. Li, Y. Yang, Z. Du, Z. Luan, X. Zhang, In situ surface-enhanced Raman scattering detection of biomolecules in the deep ocean. *Appl. Surf. Sci.* **620**, 156854 (2023).

19. L. Li, H. Zhang, S. Xi, C. Liu, C. Li, J. Zheng, J. Huang, Y. Hao, X. Zhang, W. Sun, Large hydrogen hydrothermal pipe swarm identified in the deep ocean. *Sci Bull* **70**, 2583–2586 (2025).

20. Y. Yuan, Y. Sun, S. Yan, J. Zhao, S. Liu, M. Zhang, X. Zheng, L. Jia, Multiply fully recyclable carbon fibre reinforced heat-resistant covalent thermosetting advanced composites. *Nat. Commun.* **8**, 14657 (2017).

21. S. Dasgupta, X. Peng, K. Ta, Interaction between microbes, minerals, and fluids in deep-sea hydrothermal systems. *Minerals* **11**, 1324 (2021).

22. T. Thomas, Microbial eukaryote diversity at hydrothermal vents. *Nat Rev Earth Environ* **4**, 215–215 (2023).

23. I. Srnová-Šloufová, B. Vlčková, Z. Bastl, T. L. Hasslett, Bimetallic (Ag)Au nanoparticles prepared by the seed growth method: Two-dimensional assembling, characterization by energy dispersive x-ray analysis, x-ray photoelectron spectroscopy, and surface enhanced raman spectroscopy, and proposed mechanism of growth. *Langmuir* **20**, 3407–3415 (2004).

24. S. Lee, Q. Zhao, S. Lee, I. Choi, S. Lee, I. Jung, S. Park, Two- and three-dimensional Ag–Au nanoalloying: Heterogeneous building blocks enhancing near-field focusing. *ACS Nano* **18**, 29880–29890 (2024).

25. J. Gao, N. Zhang, D. Ji, H. Song, Y. Liu, L. Zhou, Z. Sun, J. M. Jornet, A. C. Thompson, R. L. Collins, Y. Song, S. Jiang, Q. Gan, Superabsorbing metasurfaces with hybrid Ag–Au nanostructures for surface-enhanced raman spectroscopy sensing of drugs and chemicals. *Small Methods* **2**, 1800045 (2018).

26. S. Wang, B. Li, L. Li, J. Xiang, Z. Zhang, C. Wang, Z. Luan, L. Chen, X. Zhang, Flexible three-dimensional Au@Cloth SERS substrate for SERS testing of deep-sea cold seep's substances. *Chem. Eng. J.* **510**, 161592 (2025).

27. X. Yue, S. Yan, T. Gao, S. Pu, H. Tang, X. Pei, Z. Tian, X. Wang, B. Ren, G. Liu, SERS performance factor: A convenient parameter for the enhancement evaluation of SERS substrates. *Anal. Chem.* **96**, 17517–17525 (2024).

28. F. Madzharova, Z. Heiner, M. Gühlke, J. Kneipp, Surface-enhanced hyper-raman spectra of adenine, guanine, cytosine, thymine, and uracil. *J. Phys. Chem. C* **120**, 15415–15423 (2016).

29. V. Le Layec, S. Hourdez, Oxygen consumption rates in deep-sea hydrothermal vent scale worms: Effect of life-style, oxygen concentration, and temperature sensitivity. *Deep Sea Res. I Oceanogr. Res. Pap.* **172**, 103531 (2021).

30. Y.-F. Wu, M.-X. Li, J.-Z. Zhou, D.-Y. Wu, Z.-Q. Tian, Density functional theoretical study on SERS chemical enhancement mechanism of 4-mercaptopyridine adsorbed on silver. *Acta Phys-Chim Sin* **33**, 530–538 (2017).

31. J. Karlo, A. K. Dhillon, S. Siddhanta, S. P. Singh, Reverse stable isotope labelling with Raman spectroscopy for microbial proteomics. *J. Biophotonics* **17**, e202300341 (2024).

32. M.-W. Peng, X.-Y. Wei, Q. Yu, P. Yan, Y.-P. Chen, J.-S. Guo, Identification of ceftazidime interaction with bacteria in wastewater treatment by Raman spectroscopic mapping. *RSC Adv.* **9**, 32744–32752 (2019).

33. A. C. S. Talari, M. Zanyar, S. Rehman, I. U. Rehman, Raman spectroscopy of biological tissues. *Appl. Spectrosc. Rev.* **50**, 46–111 (2015).

34. P. A. Tarantilis, A. Beljebbar, M. Manfait, M. Polissiou, FT-Raman spectroscopic study of carotenoids from saffron (Crocus sativus L.) and some derivatives. *Spectrochim. Acta A Mol. Biomol. Spectrosc.* **54**, 651–657 (1998).

35. Y. Wang, J. Xu, D. Cui, L. Kong, S. Chen, W. Xie, C. Zhang, Classification and Identification of archaea Using single-cell raman ejection and artificial intelligence: Implications for investigating uncultivated microorganisms. *Anal. Chem.* **93**, 17012–17019 (2021).

36. W. E. Huang, R. I. Griffiths, I. P. Thompson, M. J. Bailey, A. S. Whiteley, Raman microscopic analysis of single microbial cells. *Anal. Chem.* **76**, 4452–4458 (2004).

37. A. Djurhuus, S.-O. Mikalsen, H.-A. Giebel, A. D. Rogers, Cutting through the smoke: The diversity of microorganisms in deep-sea hydrothermal plumes. *R. Soc. Open Sci.* **4**, 160829 (2017).

38. C. Cathalot, E. G. Roussel, A. Perhirin, V. Creff, J.-P. Donval, V. Guyader, G. Roullet, J. Gula, C. Tamburini, M. Garel, A. Godfroy, P.-M. Sarradin, Hydrothermal plumes as hotspots for deep-ocean heterotrophic microbial biomass production. *Nat. Commun.* **12**, 6861 (2021).

39. N. Kuhar, S. Sil, S. Umapathy, Potential of Raman spectroscopic techniques to study proteins. *Spectrochim. Acta A Mol. Biomol. Spectrosc.* **258**, 119712 (2021).

40. D. Cui, L. Kong, Y. Wang, Y. Zhu, C. Zhang, In situ identification of environmental microorganisms with Raman spectroscopy. *Environ. Sci. Ecotechnol.* **11**, 100187 (2022).

41. Z. Yin, R. Zheng, L. Li, S. Xi, Z. Luan, C. Sun, X. Zhang, In situ Raman quantitative monitoring of methanogenesis: Culture experiments of a deep-sea cold seep methanogenic archaeon. *Front. Microbiol.* **14**, 1128064 (2023).

42. L. Li, Z. Li, R. Zhong, Z. Du, Z. Luan, S. Xi, X. Zhang, Direct H_2S, HS^- and pH measurements of high-temperature hydrothermal vent fluids with in situ Raman spectroscopy. *Geophys. Res. Lett.* **50**, e2023GL103195 (2023).

43. M. Molari, C. Hassenrueck, R. Laso-Pérez, G. Wegener, P. Offre, S. Scilipoti, A. Boetius, A hydrogenotrophic *Sulfurimonas* is globally abundant in deep-sea oxygen-saturated hydrothermal plumes. *Nat. Microbiol.* **8**, 651–665 (2023).

44. J. Zhang, R. Liu, S. Xi, R. Cai, X. Zhang, C. Sun, A novel bacterial thiosulfate oxidation pathway provides a new clue about the formation of zero-valent sulfur in deep sea. *ISME J.* **14**, 2261–2274 (2020).

45. P. B. Dorain, J. E. Boggio, The reactions of C_2H_2 and CH_3C_2H on Ag powder. *MRS Proc.* **111**, 207 (1987).

46. A. Lisowska-Oleksiak, A. P. Nowak, M. Wilamowska, M. Sikora, W. Szczerba, C. Kapusta, Ex situ XANES, XPS and Raman studies of poly(3,4-ethylenedioxythiophene) modified by iron hexacyanoferrate. *Synth. Met.* **160**, 1234–1240 (2010).

47. G. Zhu, X. Zhu, Q. Fan, X. Wan, Raman spectra of amino acids and their aqueous solutions. *Spectrochim. Acta A Mol. Biomol. Spectrosc.* **78**, 1187–1195 (2011).

48. S. Bresson, D. Bormann, B. Khelifa, Raman studies of the C–H stretching modes in various cholesteryl alkanoates. *Vib. Spectrosc.* **16**, 163–171 (1998).

49. N. K. Howell, G. Arteaga, S. Nakai, E. C. Y. Li-Chan, Raman spectral analysis in the C−H stretching region of proteins and amino acids for investigation of hydrophobic interactions. *J. Agric. Food Chem.* **47**, 924–933 (1999).

50. H. Schulz, M. Baranska, Identification and quantification of valuable plant substances by IR and Raman spectroscopy. *Vib Spectrosc* **43**, 13–25 (2007).

51. M. Li, H. Chen, M. Wang, Z. Zhong, C. Lian, L. Zhou, H. Zhang, H. Wang, L. Cao, C. Li, Phenotypic plasticity of symbiotic organ highlight deep-sea mussel as model species in monitoring fluid extinction of deep-sea methane hydrate. *Sci. Total Environ.* **958**, 178048 (2025).

52. Z. Xu, M. Wang, H. Zhang, W. He, L. Cao, C. Lian, Z. Zhong, H. Wang, L. Fu, X. Zhang, C. Li, Metabolism interactions promote the overall functioning of the episymbiotic chemosynthetic community of *Shinkaia crosnieri* of cold seeps. *mSystems* **7**, e00320–e00322 (2022).

53. R. B. van Breemen, C.-R. Huang, Y. Tan, L. C. Sander, A. B. Schilling, Liquid chromatography/mass spectrometry of carotenoids using atmospheric pressure chemical ionization. *J. Mass Spectrom.* **31**, 975–981 (1996).

54. Y. Han, C. Zhang, Z. Zhao, Y. Peng, J. Liao, Q. Jiang, Q. Liu, Z. Shao, X. Dong, A comprehensive genomic catalog from global cold seeps. *Sci. Data* **10**, 596 (2023).

55. T. Seemann, Prokka: Rapid prokaryotic genome annotation. *Bioinformatics* **30**, 2068–2069 (2014).

56. J. Mistry, S. Chuguransky, L. Williams, M. Qureshi, G. A. Salazar, E. L. L. Sonnhammer, S. C. E. Tosatto, L. Paladin, S. Raj, L. J. Richardson, R. D. Finn, A. Bateman, Pfam: The protein families database in 2021. *Nucleic Acids Res.* **49**, D412–D419 (2021).

57. P. A. Chaumeil, A. J. Mussig, P. Hugenholtz, D. H. Parks, GTDB-Tk v2: Memory friendly classification with the genome taxonomy database. *Bioinformatics* **38**, 5315–5316 (2022).

58. M. N. Price, P. S. Dehal, A. P. Arkin, FastTree 2—Approximately maximum-likelihood trees for large alignments. *PLOS ONE* **5**, e9490 (2010).

59. Y. Liu, H. Zhang, L. Cao, L. Fu, C. Lian, Y. Guo, Z. Zhong, I. Seim, M. Wang, C. Li, Methane filtration and metabolic cooperation of microbial communities in cold seep water columns from South China Sea. *Commun. Biol.* **8**, 1052 (2025).

60. G. Yuan, D. Liu, X. Feng, M. Shao, Z. Hao, T. Sun, H. Yu, H. Ge, X. Zuo, Y. Zhang, In situ fabrication of porous Co_xP hierarchical nanostructures on carbon fiber cloth with exceptional performance for sodium storage. *Adv. Mater.* **34**, e2108985 (2022).

61. N. Matsuhisa, D. Inoue, P. Zalar, H. Jin, Y. Matsuba, A. Itoh, T. Yokota, D. Hashizume, T. Someya, Printable elastic conductors by in situ formation of silver nanoparticles from silver flakes. *Nat. Mater.* **16**, 834–840 (2017).

62. A. Sakthisabarimoorthi, S. A. M. B. Dhas, M. Jose, Preparation of composite Ag@Au core–shell nanoparticles and their linear and nonlinear optical properties. *J. Mater. Sci. Mater. Electron.* **30**, 1677–1685 (2019).

63. M.-S. Hu, H.-L. Chen, C.-H. Shen, L.-S. Hong, B.-R. Huang, K.-H. Chen, L.-C. Chen, Photosensitive gold-nanoparticle-embedded dielectric nanowires. *Nat. Mater.* **5**, 102–106 (2006).

64. H.-L. Wu, L. A. Huff, A. A. Gewirth, In situ Raman spectroscopy of sulfur speciation in lithium–sulfur batteries. *ACS Appl. Mater. Interfaces* **7**, 1709–1719 (2015).

65. W. He, R. Cai, S. Xi, Z. Yin, Z. Du, Z. Luan, C. Sun, X. Zhang, Study of microbial sulfur metabolism in a near real-time pathway through confocal Raman quantitative 3D Imaging. *Microbiol. Spectrum* **11**, e03678–e03622 (2023).
